# Furfuryl Alcohol‐Driven Proton Supply Enables Efficient Photocatalytic H_2_O_2_ Production Beyond Water‐Based Systems

**DOI:** 10.1002/advs.76085

**Published:** 2026-06-11

**Authors:** Pengfei Bai, Ning Li, Quan Zhou, Bin Liu, Xiangqian Fan, Lei Liu, Jingkai Lin, Weijie Ren, Shengliang Hu, Huayang Zhang

**Affiliations:** ^1^ School of Energy and Power Engineering & State Key Laboratory of Coal and CBM Co‐Mining North University of China Taiyuan China; ^2^ School of Chemical Engineering Adelaide University Adelaide SA Australia; ^3^ Shanxi Center of Technology Innovation for Light Manipulations and Applications School of Applied Science Taiyuan University of Science and Technology Taiyuan China

**Keywords:** built‐in electric field, charge separation, furfuryl alcohol oxidation, photocatalytic H_2_O_2_ production, proton supply, ZnIn_2_S_4_/carbon dots

## Abstract

Photocatalytic H_2_O_2_ production via the two‐electron oxygen reduction reaction is often limited by rapid charge recombination and insufficient proton supply in aqueous systems. Here, we construct a covalently linked ZnIn_2_S_4_‐carbon dots (ZIS‐CDs) develop a coupled system that integrates H_2_O_2_ generation with selective oxidation of furfuryl alcohol (FFA). The built‐in electric field in the ZIS‐CDs heterojunction enables efficient charge separation. FFA acts simultaneously as a sacrificial electron donor, oxidation substrate, and proton source in the ultra‐dry acetonitrile (ACN)‐FFA system. During its oxidation, FFA supplies protons for H_2_O_2_ generation and is selectively converted into furoic acid (FA). This dual regulation of charge transfer and proton supply leads to an H_2_O_2_ production rate of 24 mmol·g^−^^1^·h^−^^1^, an apparent quantum yield of 14.57% at 400 nm, and a solar‐to‐chemical conversion efficiency of 1.78%, with 100% selectivity and 98% conversion to furoic acid. This work introduces a new strategy that couples organic transformation with proton management to enhance photocatalytic H_2_O_2_ synthesis.

## Graphical abstract

A ZnIn_2_S_4_–carbon dot heterojunction enables photocatalytic H_2_O_2_ production coupled with furfuryl alcohol oxidation. In this redox system, furfuryl alcohol acts as both the electron donor and oxidation substrate, producing furoic acid with 100% selectivity and 98% conversion while supporting efficient H_2_O_2_ generation with a solar‐to‐chemical conversion (SCC) efficiency of 1.78%.

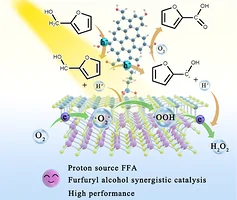

## 1 Introduction

Hydrogen peroxide (H_2_O_2_) is a crucial green oxidant extensively employed in fuel processing, wastewater treatment, bleaching, and disinfection [1, 2, 3, 4, 5]. Industrial production mainly relies on the anthraquinone process, which involves sequential hydrogenation and oxidation steps under energy‐intensive conditions. The complexity and environmental concerns associated with this process have driven the search for sustainable alternatives. Photocatalytic H_2_O_2_ production, which directly converts solar energy into chemical energy, is considered a promising approach [6, 7, 8, 9, 10]. In typical photocatalytic systems, semiconductors such as ZnIn_2_S_4_, In_2_S_3_, MoS_2_, ZnO, C_3_N_4_, covalent organic frameworks, metal‐organic frameworks, and carbon dots(CDs) drive two half‐reactions: the two‐electron oxygen reduction reaction (2e^−^ ORR) and the water oxidation reaction (WOR) [4, 5, 6, 7, 8, 11, 12, 13, 14]. Photogenerated electrons reduce O_2_ to H_2_O_2_ (O_2_+ 2H^+^+ 2e^−^ → H_2_O_2_), while photogenerated holes promote the WOR to oxidize H_2_O to H_2_O_2_ (2 H_2_O + 2h^+^ → H_2_O_2_ + 2H^+^) [8]. The two‐electron WOR pathway (H_2_O→H_2_O_2_, 1.76 V vs. NHE) typically demands a higher redox potential than the two‐electron ORR pathway (O_2_→H_2_O_2_, 0.695 V vs. NHE), and its sluggish reaction kinetics lead to excessive accumulation of photogenerated holes, further exacerbating charge recombination and decreasing the photocatalytic performance [8, 12].

To improve performance, most studies have focused on enhancing charge separation through heterojunction construction, defect engineering, and cocatalyst modification [6, 7, 8, 15, 16, 17, 18, 19]. Nevertheless, photocatalytic H_2_O_2_ production fundamentally proceeds through proton‐coupled electron transfer (PCET) [20, 21]. Besides electrons, interfacial protons also play a pivotal role in the kinetics of PCET. In existing aqueous catalytic systems, the protons required for the 2e^−^ ORR to generate H_2_O_2_ are mainly derived from water dissociation. This process is kinetically sluggish due to its high energy barrier and the low proton diffusion coefficient in water (∼2.39 × 10^−^^9^ m^2^/s at 300 K) [22], which results in a prevalent proton‐deficient microenvironment around the catalyst and directly limits the total H_2_O_2_ yield. To address these issues, strategies including the protonation of functional units and the introduction of strongly polar oxygen‐containing functional groups (e.g., ─SO_3_H, ─COOH) have been developed to increase the proton concentration in the system [23]. For example, Zhu et al. prepared protonated hydrogen‐bonded organic frameworks (HCOFs) via sulfuric acid acidification. Protonation significantly extended the light absorption range to ∼700 nm, thus improving the photocatalytic H_2_O_2_ production and achieving a high apparent quantum efficiency (AQY) of 2.05% at 600 nm [24]. Li et al. uniformly immobilized ─SO_3_H or ─COOH groups onto highly crystalline triazine‐based COFs via a topology‐guided synthesis strategy [25]. These groups acted as “dual‐functional electron/proton extractors”, which not only enhances the electron extraction capacity but also facilitates proton transport through hydrogen‐bonding networks, thereby realizing the synergistic acceleration of the PCET process. Despite the advances achieved, each approach has its own inherent limitations. For instance, organic materials tend to hydrolyze under protonation conditions, and introducing oxygen‐containing functional groups often requires cumbersome procedures, which hampers the overall efficiency of photosynthetic H_2_O_2_ production. Therefore, the construction of novel synergistic catalytic coupling systems is of great research significance.

In recent years, researchers have incorporated selective organic oxidation reactions to rapidly scavenge photogenerated holes, while utilizing photogenerated electrons for the 2e^−^‐ORR process, thereby achieving the co‐production of H_2_O_2_ and high‐value organic compounds [26, 27, 28, 29, 30, 31]. Furoic acid (FA) is a critical intermediate for the synthesis of plasticizers, thermosetting resins, preservatives, coating additives, and other products [32]. The oxidation of furfural alcohol (FFA) to FA releases a considerable number of protons. Directly utilizing these protons to drive 2e^−^‐ORR for H_2_O_2_ generation synergistically accelerates the PCET process. Thus, the photocatalytic production of H_2_O_2_ coupled with the selective oxidation of FFA to FA exhibits substantial potential.

**SCHEME 1:**
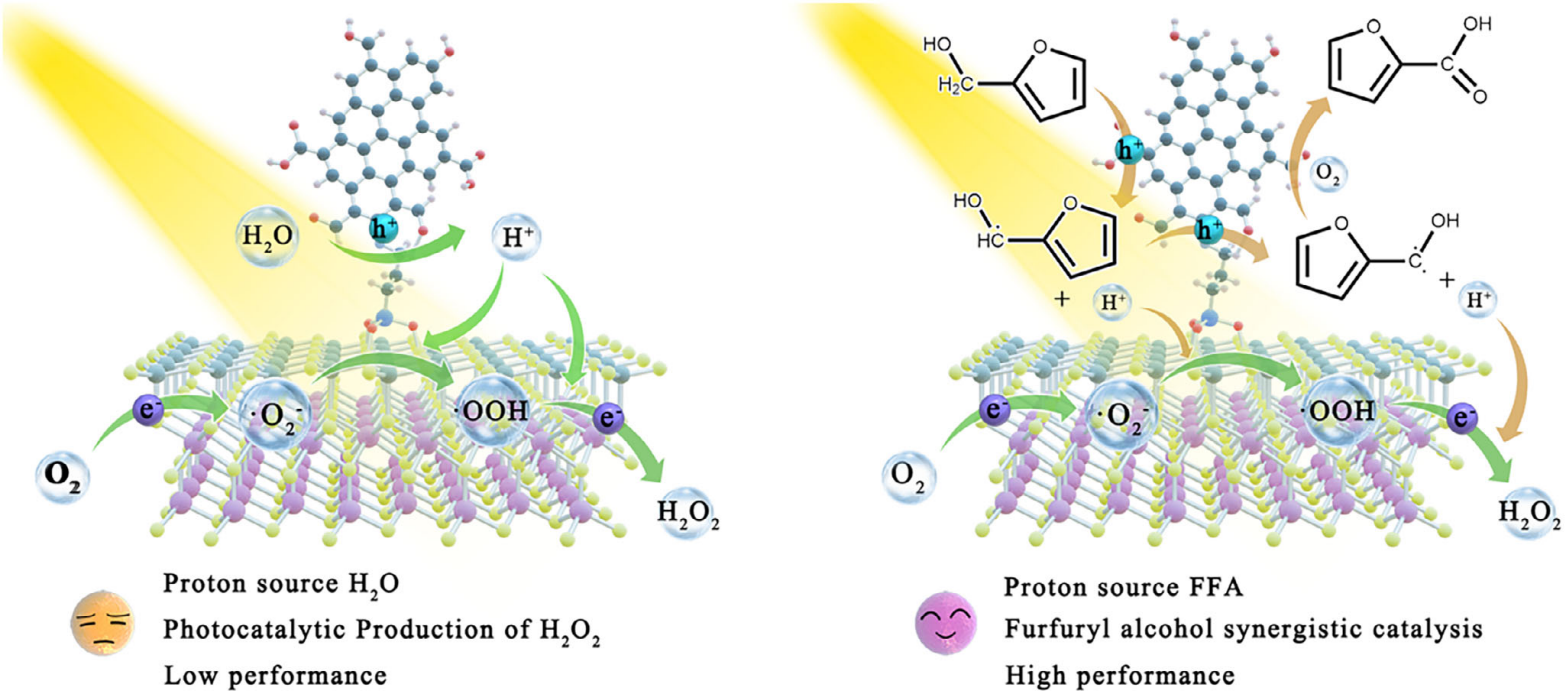
Schematic diagram of photocatalytic H_2_O_2_ production assisted by different proton sources originated from water dissociation and FFA photooxidation.

In this work, we coupled FFA conversion with photocatalytic H_2_O_2_ synthesis in an acetonitrile (ACN)/FFA catalytic system, attaining a maximum H_2_O_2_ production rate of 24 mmol·g^−1^·h^−1^. This study innovatively proposes a novel system of ‘photocatalytic oxygen reduction coupled with FFA photooxidation’. By using the ZIS‐CDs heterojunction to promote charge separation and interfacial electron transfer, FFA oxidation was coupled with photocatalytic H_2_O_2_ production to form an integrated redox system (Scheme 1). In conventional aqueous photocatalytic H_2_O_2_ production, protons are mainly derived from water. In contrast, in the ACN‐FFA system, FFA functions as both a sacrificial electron donor and a major proton source. FFA reacts with photogenerated holes to release protons for H_2_O_2_ formation, while being selectively converted into FA. This strategy integrates proton supply with target product synthesis, achieving 100% selectivity and 98% conversion toward FA. Theoretical calculations demonstrate that the efficient electron transfer channels constructed by ZIS‐CDs facilitate charge separation and transfer, substrate activation, and the dehydrogenation process of FFA. These effects further accelerate the reaction kinetics of both H_2_O_2_ production and FFA photooxidation, thereby enhancing the overall reaction efficiency. This work offers a promising and innovative research direction for advancing sustainable photocatalytic H_2_O_2_ generation technology.

## 2 Results and Discussion

### 2.1 Catalyst Synthesis, Structure, Morphology and Composition Characterization

**FIGURE 1:**
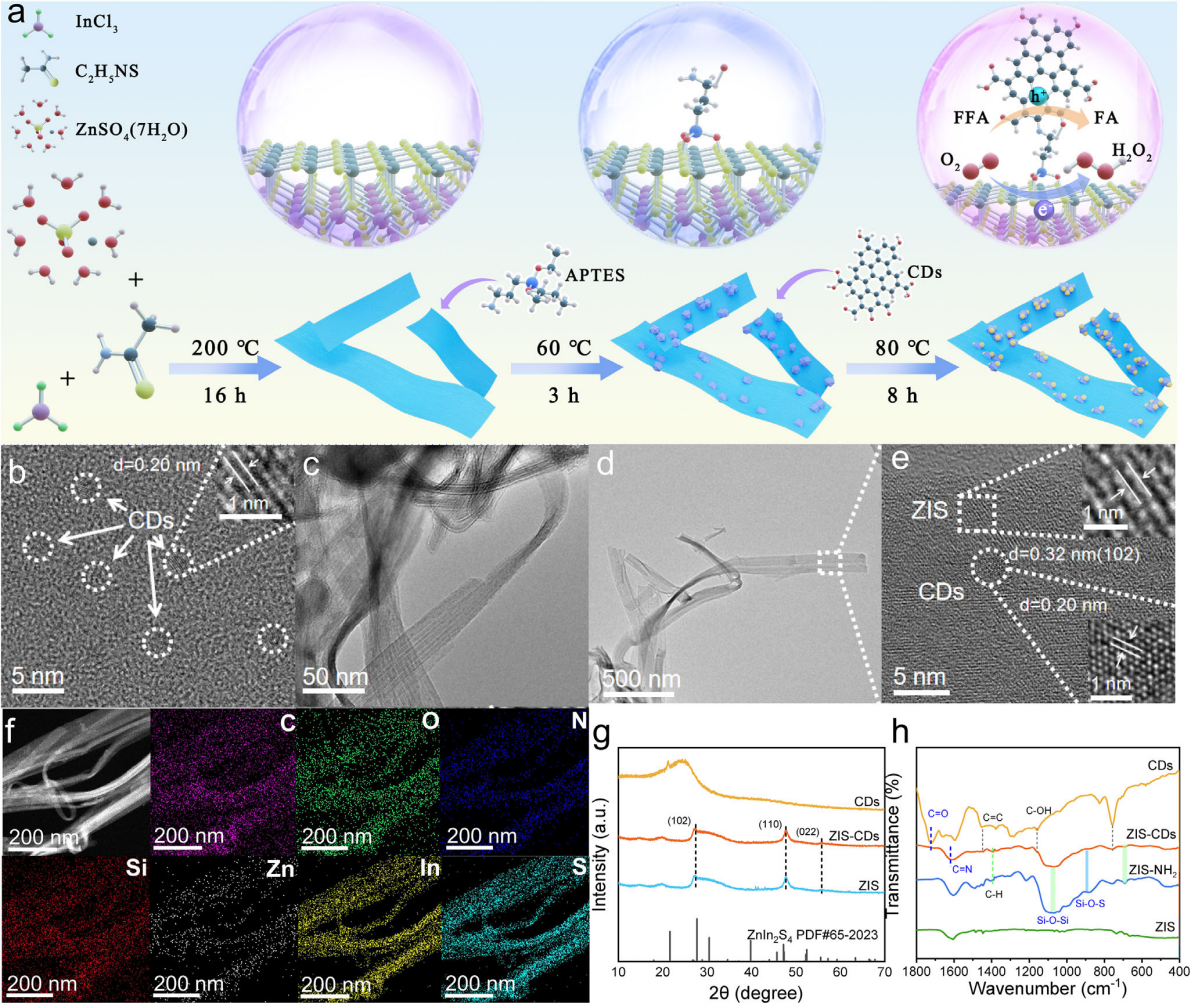
(a) Schematic diagram of covalently grafting CDs molecules onto ZnIn2S4 nanobelts via Schiff base polycondensation. TEM and high‐resolution TEM images of (b) CDs (c) ZIS, and (d, e) ZIS‐CDs. (f) Element mapping images of C, N, O, Si, In, Zn, and S for ZIS‐CDs sample measured by STEM‐EDS. (g) XRD patterns of ZIS, ZIS‐CDs, and CDs molecules. (h) FTIR spectra of ZIS, ZIS‐NH2, ZIS‐CDs, and CDs molecules.

Figure 1a shows the fabrication of the ZIS‐CDs heterojunction. In brief, carbon dots were covalently anchored onto ZnIn_2_S_4_(ZIS) nanobelts through APTES‐assisted Schiff‐base coupling, forming a tightly bonded heterostructure. The microstructures of CDs, ZIS, and ZIS‐CDs photocatalysts were observed by using transmission electron microscopy (TEM). As displayed in Figure 1b and Figure S1, CDs are uniformly dispersed, with a lattice spacing of 0.20 nm, corresponding to the (101) crystal plane of the graphite phase [33]. Both ZIS and the ZIS‐CDs hybrid material exhibit typical 1D nanobelt morphologies with consistent structural regularity (Figure 1c,d). This observation indicates that the introduction of CDs molecules only modifies the surface of ZIS without altering its intrinsic morphology, demonstrating good structural compatibility during the hybridization process. In Figure 1e, two distinct lattice spacings can be observed. The lattice fringe spacing of 0.32 nm can be indexed to the (102) crystal plane of ZnIn_2_S_4_, whereas a measured interplanar distance of 0.20 nm is characteristic of the (101) crystal plane of CDs, consistent with previous literature reports [33, 34]. Furthermore, the energy dispersive spectroscopy (EDS) elemental mapping (Figure 1f) reveals a uniform distribution of Mo, Zn, In, S, C, O, Si, and N elements in the ZIS‐CDs heterostructure. This further verifies that the CDs are uniformly dispersed on the surface of the ZIS nanobelts, laying a structural foundation for the highly efficient photocatalytic performance of this catalyst. X‐ray diffraction (XRD) patterns were performed to analyze the crystallographic information of the samples (Figure 1g). The characteristic diffraction peaks of both ZIS and ZIS‐CDs samples at 21.6°, 27.7°, 30.4°, 39.8°, 47.2°, 52.4°, and 55.6°can all be attributed to the hexagonal phase of ZIS (JCPDS: 65–2023) [35, 36], indicating that the introduction of CDs does not alter the integrity of the ZIS crystal structure. The characteristic diffraction signals of CDs are not observed, primarily due to the low content and their highly uniform dispersion on the ZIS surface [37]. As a result, their diffraction signals are masked by the strong characteristic peaks of ZIS.

To verify the covalent bonding between ZIS and CDs, Fourier transform infrared (FTIR) spectra of ZIS, ZIS‐NH_2_, CDs, and ZIS‐CDs were measured (Figure 1h, Figure S2). The ZIS nanobelts exhibit a broad absorption peak in the range of 3200–3600 cm^−1^, indicative of the ─OH stretching vibration, and a peak at 1401 cm^−1^ is attributed to the ─OH bending vibration [38], which provides the necessary reactive sites for APTES functionalization. Compared to pure ZIS, the FTIR spectrum of ZIS‐NH_2_ shows several new characteristic peaks. The peaks at 2925 and 1385 cm^−1^ are assigned to the stretching and bending vibrations of the Si─CH_2_ group in APTES molecules, respectively [39]. Peaks in the 600–800 nm^−1^ range and at 1116 cm^−1^ belong to the asymmetric stretching and bending vibrations of the siloxane group (Si─O─Si) [40]. The peak at 890 cm^−1^ is ascribed to the stretching vibration of the Si─O─S bond [41]. These characteristic peaks confirm the successful grafting of APTES onto the ZIS surface via chemical bonding, achieving ZIS‐NH_2_. Compared to the ZIS‐NH_2_ sample, the ZIS‐CDs sample retains its key characteristic peaks while displaying additional peaks belonging to CDs. Importantly, C═O vibrational peak of the ZIS‐CDs is significantly reduced compared to that of the CDs [42], which indicates CDs molecules successfully graft onto the ZIS surface via Schiff‐base reaction.

X‐ray photoelectron spectroscopy (XPS) was employed to systematically investigate the elemental composition and chemical states of the as‐prepared samples. As presented in the XPS survey spectrum (Figure S3a), the characteristic signals of C, N, O, S, In, and Zn elements are clearly distinguishable, which is in good agreement with the results obtained from EDS element mapping. This consistency further corroborates the successful incorporation of APTES and CDs into the ZIS‐CDs hybrid system. The high‐resolution N 1s spectrum (Figure S3b) was deconvoluted into two distinct peaks centered at 399.8 and 401.70 eV, which can be assigned to the C═N bond and other nitrogen‐containing species, respectively [35]. Notably, the presence of the C═N bond provides direct evidence that CDs are covalently anchored to ZIS‐NH_2_ via a Schiff base reaction, which is crucial for constructing a stable interfacial contact between the two components. The high‐resolution XPS spectra of C 1s, Zn 2p, In 3d, and S 2p are displayed in Figure S3c–f, respectively, to gain deeper insights into the chemical bonding configurations of the ZIS‐CDs heterojunction. For the C 1s spectrum, three characteristic peaks at 284.71, 286.30, and 288.20 eV are observed, corresponding to the C─C, C─O/C─N, and C═O bonds in the ZIS‐CDs sample [43]. Intriguingly, a noticeable shift of the C 1s peaks toward lower binding energies is observed when compared with the pure CDs sample. In the Zn 2p spectrum of ZIS‐CDs, two well‐resolved peaks at 1045.05 and 1022.01 eV are identified, which are characteristic of Zn 2p_1/2_ and Zn 2p_3/2_ orbitals, respectively [44]. Similarly, the In 3d spectrum exhibits two distinct peaks at 452.55 and 445.01 eV, which are attributed to In 3d_3/2_ and In 3d_5/2_, respectively. The S 2p spectrum can be deconvoluted into a spin‐orbit doublet with binding energies of 162.75 and 161.55 eV, corresponding to S 2p_1/2_ and S 2p_3/2_ in the ZIS‐CDs sample, respectively. In sharp contrast to the ZIS‐NH_2_ sample, the binding energies of Zn 2p, In 3d, and S 2p of ZIS‐CDs exhibit significant positive shifts, which is contrary to that of the C 1s. This phenomenon demonstrates that the ZIS‐CDs interface possesses strong interfacial interaction, with electrons spontaneously migrating from ZIS‐NH_2_ to CDs due to the differences in Fermi energy levels (*E*_f_) [40], resulting in the generation of a built‐in electric field at their interface [35]. The Brunauer–Emmett–Teller (BET) specific surface area and pore structure of the samples were characterized via N_2_ adsorption‐desorption (Figure S4). The specific surface area of pristine ZIS was measured to be 111.14 m^2^/g, while that of the ZIS‐CDs heterojunction decreased to 75.28 m^2^/g. The reduction in surface area can be attributed to the close contact formed between CD molecules and the ZIS surface via covalent bonding, which partially blocks the pores on ZIS and reduces its effective adsorption area [44].

### 2.2 Photocatalytic Performance Evaluation

**FIGURE 2:**
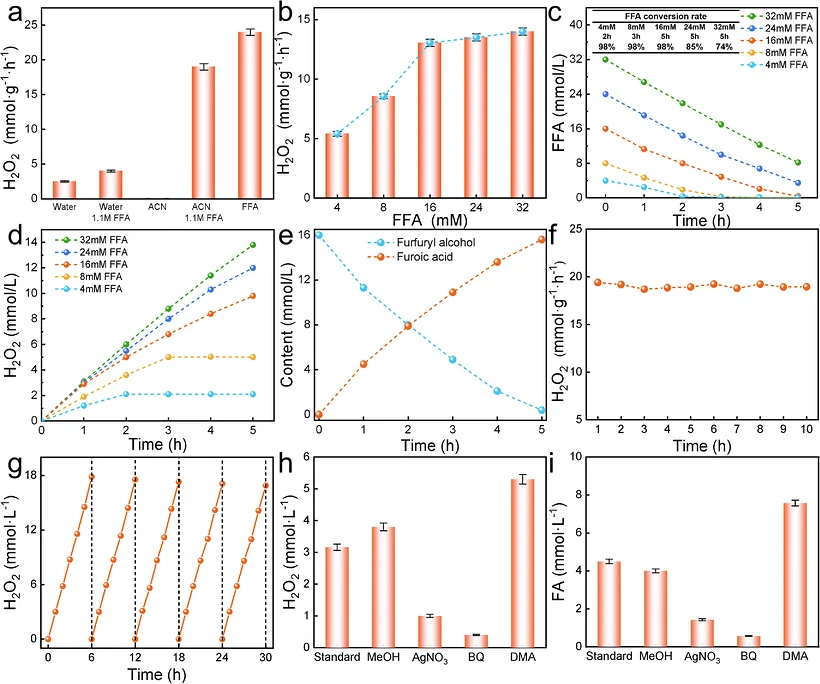
(a) H_2_O_2_ yield of ZIS‐CDs under different solvents. (b) Photocatalytic H_2_O_2_ production rate of ZIS‐CDs at different FFA concentrations within 1 h. (c) Time‐dependent FFA concentration profiles over ZIS‐CDs at various FFA concentrations. (d) Time‐dependent H_2_O_2_ concentration profiles over ZIS‐CDs at various FFA concentrations. (e) Temporal evolution of FFA and FA concentrations over ZIS‐CDs in 16 mm FFA solution. (f) Photocatalytic H_2_O_2_ evolution stability of ZIS‐CDs in 1.1 m FFA aqueous solution. (g) Photocatalytic H_2_O_2_ production cycle test over ZIS‐CDs under 1.1 m FFA. (h, i) Active species trapping experiments for ZIS‐CDs.

As depicted in Figure S5a, the pristine ZIS photocatalyst exhibits an H_2_O_2_ production rate of 1.1 mmol·g^−1^·h^−1^ in aqueous solution. After compositing ZIS with CDs at varying mass ratios, the ZIS‐CDs composite with a mass ratio of 5:1 (denoted as ZIS‐CDs‐5:1) demonstrates the optimal photocatalytic performance for H_2_O_2_ production. To clarify the reaction pathway, the catalytic performance of the ZIS‐CDs‐5:1 sample was further compared under an O_2_‐saturated atmosphere versus evacuated conditions. The H_2_O_2_ production rate increased significantly in the O_2_‐saturated environment, whereas it dropped to nearly zero under vacuum. These results confirm that the photocatalytic H_2_O_2_ generation over the ZIS‐CDs heterojunction follows a sole ORR pathway, and the participation of molecular oxygen is an essential prerequisite for H_2_O_2_ formation in this system. Subsequently, this study designed and conducted coupled reactions for photocatalytic H_2_O_2_ production and FFA photo‑oxidation under varied conditions. Using 5 mg of ZIS‑CDs as the photocatalyst dispersed in 30 mL of different reaction solutions, the reactions were carried out under continuous O_2_ bubbling, visible light (λ > 420 nm) and 15°C, with a focus on the effects of solution type and FFA concentration on the H_2_O_2_ yield. As shown in Figure 2a, H_2_O_2_ was generated only at a low rate of 2.4 mmol·g^−1^·h^−1^ in the aqueous system alone. To investigate the role of oxygen in the photocatalytic production of H_2_O_2_, H_2_O_2_ photosynthesis experiments were carried out using oxygen‐saturated aqueous solution. The results demonstrated that when ZIS‐CDs served as the catalyst, the H_2_O_2_ generation rate was enhanced up to 3.3 mmol·g^−1^·h^−1^ (Figure S5b). In sharp contrast, the H_2_O_2_ yield decreased significantly under vacuum conditions, which fully confirmed that oxygen is an essential reactant for the H_2_O_2_ generation reaction in this photocatalytic system. When 10 vol% FFA was added to the water as a scavenger, the H_2_O_2_ production rate increased to 3.5 mmol·g^−1^·h^−1^. In aqueous systems, water molecules as proton donors exhibit a relatively low proton release rate, which is insufficient to meet the continuous proton demand for photocatalytic H_2_O_2_ synthesis, thereby limiting efficient H_2_O_2_ production [45, 46, 47]. To address this issue, FFA is employed in this work to act simultaneously as a sacrificial electron donor and oxidation substrate. To eliminate the interference of water, ACN was introduced as the reaction solvent. Experimental results confirm that no H_2_O_2_ is detected in pure ACN (Figure 2a), indicating that ACN neither supports H_2_O_2_ production over ZIS‐CDs nor provides protons. This excludes the contribution of ACN to proton supply and confirms that ACN mainly serves as a non‐protic solvent and diluent for preparing the ACN/FFA system. In Figure 2a, when ACN was used as the diluent, the H_2_O_2_ production rate reached 19 mmol g^−1^ h^−1^ at an FFA concentration of 1.1 m (approximately 10 vol%), while the maximum activity of 24 mmol g^−1^ h^−1^ was achieved in neat FFA. To clarify the role of FFA concentration, H_2_O_2_ evolution was first evaluated within 1 h using FFA concentration as the only variable. As presented in Figure 2b, the H_2_O_2_ production rate increased with FFA concentration. Time‐dependent measurements were then performed over 5 h at different FFA concentrations. Low‐concentration FFA was gradually consumed during the reaction (Figure 2c), leading to a clear decrease in H_2_O_2_ generation over time (Figure 2d). Further analysis using 16 mm FFA showed that FFA was almost completely consumed after 5 h, with 98% conversion and nearly 100% selectivity toward FA (Figure 2e and Figure S6). Because dilute FFA cannot support stable long‐term H_2_O_2_ production, 1.1 m FFA/ACN solution was used for the stability test. Under this condition, the H_2_O_2_ evolution rate remained nearly constant over 10 h (Figure 2f). These results manifest that FFA can not only effectively accelerate the photosynthetic production of hydrogen peroxide, but also undergo highly selective conversion into FA. By analyzing the coupled reaction data at different FFA concentrations, the experimental molar ratio between H_2_O_2_ evolution and FFA oxidation was determined to be approximately 0.7:1 across all tested systems. This ratio confirms the close correlation between FFA oxidation and H_2_O_2_ formation, while the deviation from the theoretical 1:1 ratio may arise from side reactions, incomplete charge utilization, and competitive consumption of reactive intermediates during the coupled photocatalytic process.

The apparent quantum yields (AQY) of ZIS‐CDs were determined to be 14.57%, 13.8%, and 12.5% at 400, 420, and 500 nm, respectively (Figure S7a, Table S1), which is in good agreement with the UV–vis absorption spectrum of the ZIS‐CDs composite. Solar‐to‐chemical conversion (SCC) efficiency was calculated as 1.78% (Figure S7b), indicating that ZIS‐CDs possess efficient photon‐to‐chemical energy conversion capability in the UV‐visible region for photocatalytic H_2_O_2_ production. Cycling tests of ZIS‐CDs over five consecutive cycles in 1.1 m FFA/CAN solution showed no obvious decrease in H_2_O_2_ production activity or FFA conversion efficiency (Figure 2g and Figure S8a). TEM images confirmed that the morphology of ZIS‐CDs remained well preserved after the reaction (Figure S8b). The characteristic peaks in FTIR spectra and XRD patterns showed no obvious changes after cycling, confirming the stable chemical bonding and crystal structures of the catalyst (Figure S8c,d). These results demonstrate the good cycling stability of the ZIS‐CDs catalyst. Comparative analysis shows that the ZIS‐CDs heterojunction exhibits superior photocatalytic H_2_O_2_ production and FFA conversion performances over numerous other catalysts (Figure S7b, Tables S2 and S3) [3, 4, 6, 7, 8, 9, 11, 12, 13, 15, 16, 17, 18, 19, 26, 29, 30, 35, 43].

### 2.3 Intrinsic Mechanism Underlying the Efficient Photocatalytic Performance

To elucidate the cooperative mechanism between photocatalytic H_2_O_2_ production and FFA‐to‐FA conversion, this study systematically investigated the roles of key reactive intermediates through active species trapping experiments and electron paramagnetic resonance (EPR) spectroscopy. First, specific scavengers were introduced into the reaction system to selectively quench potential active species generated during photocatalysis. The effects on both H_2_O_2_ yield and FA production were examined to clarify the contribution of each species (Figure 2h,i). The scavengers and their targets were as follows: AgNO_3_ (to trap photogenerated electrons), benzoquinone (BQ, to quench superoxide radicals ·O_2_^−^), methanol (MeOH, to scavenge photogenerated holes), and dimethyl anthracene (DMA, to quench singlet oxygen ^1^O_2_). The addition of AgNO_3_ or BQ significantly suppressed both H_2_O_2_ and FA productions, confirming that photogenerated electrons and ·O_2_^−^ are key active radicals driving the coupled synthesis of H_2_O_2_ and oxidation of FFA [28, 29, 30, 31]. When MeOH was introduced, the H_2_O_2_ generation rate increased, while FA yield decreased, indicating that holes participated in FFA oxidation. Interestingly, the incorporation of DMA induced a remarkable enhancement in the generation rates of H_2_O_2_ and FA, with the values increasing to 33 and 72 mmol·g^−^^1^·h^−^^1^, respectively. Subsequent EPR analysis of the ACN+FFA system showed no characteristic signal of ^1^O_2_ (Figure S9a). A control experiment using benzylamine instead of FFA yielded highly consistent results upon DMA addition (Figure S10). Combining these observations, DMA does not function as a ^1^O_2_ quencher in this system. Instead, its primary role is to promote the efficient transfer of photogenerated electrons [29], thereby accelerating the generation and transport kinetics of ·O_2_^−^ and holes. This enhancement strengthens the synergy between H_2_O_2_ synthesis and organic substrate oxidation, ultimately driving the overall photocatalytic cooperative reaction with high efficiency.

**FIGURE 3:**
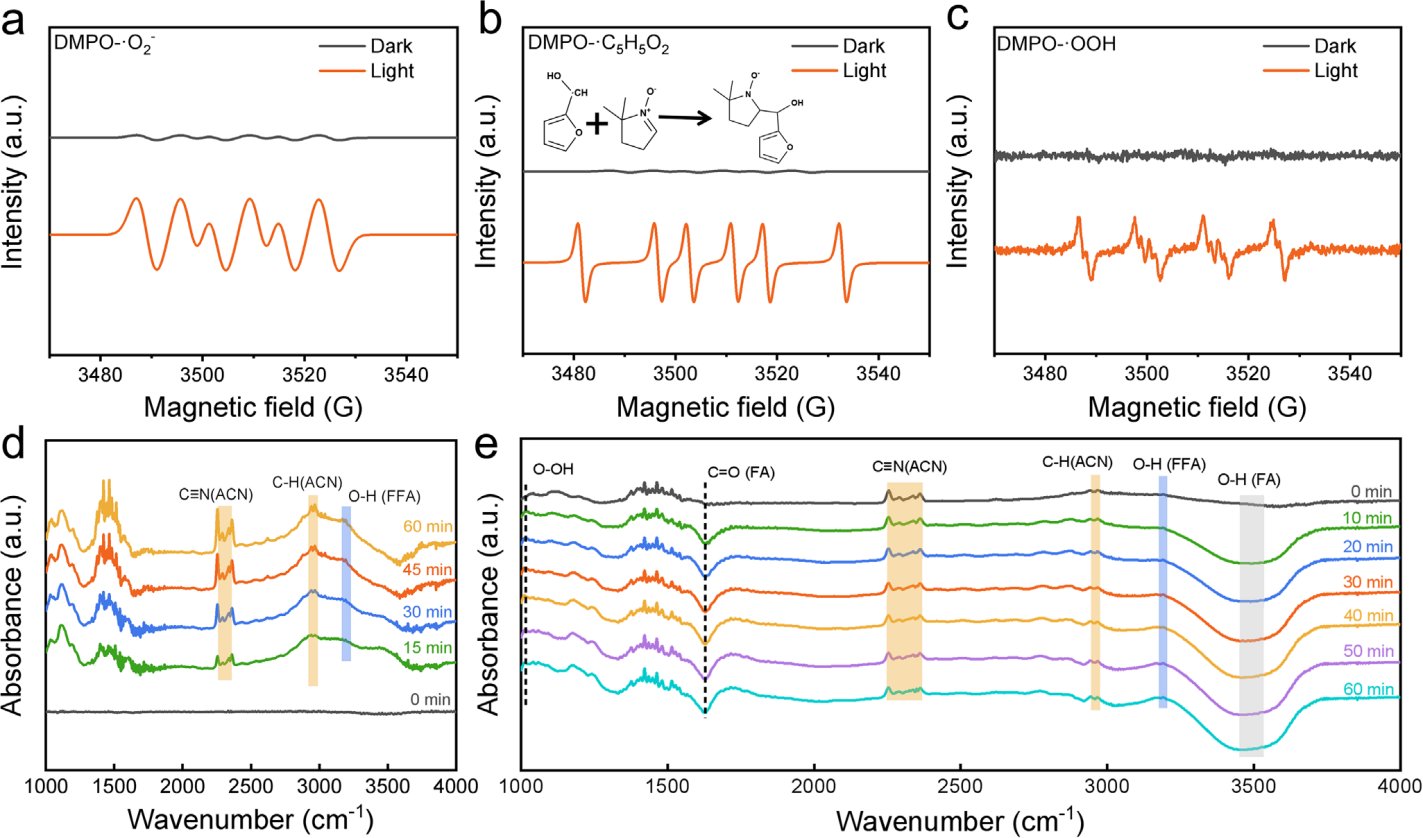
(a) EPR signals of DMPO‐·O_2_ on ZIS‐CDs in O_2_‐saturated furfuryl alcohol. (b) EPR signals of DMPO‐·C_5_H_5_O_2_ over ZIS‐CDs in FFA solution in dark and under light irradiation. (c) EPR signals of DMPO‐ ·OOH on ZIS‐CDs in O_2_‐saturated furfuryl alcohol; In situ DRIFTS spectra of ZIS‐CDs exposed to ACN+FFA system (d) in the dark (e) under illumination.

Electron paramagnetic resonance (EPR) spectroscopy was employed to discern the existence and transformation of active species and intermediates [48, 49]. Using 5, 5‐dimethyl‐1‐pyrroline N‐oxide (DMPO) and 2,2,6,6‐tetramethylpiperidine (TEMP) as trapping agents, red under light irradiation. As shown in Figure S9b, no signals corresponding to ·OH were detected for the ZIS‐CDs sample under illumination. Importantly, characteristic signals of ·O_2_^−^ and the intermediate radical ·C_5_H_5_O_2_ were clearly observed after 10 min of light exposure. Concurrently (Figure 3a,b), signals of hydroperoxyl radicals (·OOH) were also detected (Figure 3c).·OOH and ·C_5_H_5_O_2_ are the key intermediates formed via the protonation of ·O_2_^−^ and the conversion of FFA. These observations indicate that the ZIS‐CDs heterojunction facilitates the photocatalytic reduction of O_2_ to H_2_O_2_ alongside the conversion of FFA to FA.

To evaluate the ORR tendency of ZIS‐CDs, the average electron transfer number during O_2_ reduction was estimated from linear sweep voltammetry (LSV) curves obtained using rotating ring‐disk electrode (RRDE) measurements and the Koutecky–Levich (K‐L) equation (Figure S11a–c) [50]. The electron transfer number increased from 1.25 to 1.43 after adding FFA, suggesting an enhanced tendency toward the 2e^−^ ORR pathway. Because RRDE measurements do not directly represent the photocatalytic reaction environment, gas‐controlled photocatalytic experiments were further performed in 1.1 m FFA/ACN solution under different atmospheres at the same flow rate (Figure S11d). The H_2_O_2_ production rate was highest under pure O_2_ and gradually decreased when O_2_ was diluted with air or Ar. Only limited H_2_O_2_ production was observed under pure Ar or N_2_, confirming that molecular O_2_ is the primary electron acceptor and that photocatalytic H_2_O_2_ generation mainly proceeds through the ORR pathway.

To investigate the surface‐adsorbed species and reaction intermediates, in situ diffuse reflectance infrared Fourier transform spectroscopy (DRIFTS) was performed. When the ZIS‐CDs sample was exposed to a vapor mixture of FFA, acetonitrile, and O_2_ in the dark for 1 h, characteristic absorption peaks corresponding to FFA and ACN were detected, with their intensities gradually increasing over time. As shown in Figure 3d, the absorption peak at 3270 cm^−1^ can be assigned to the O─H stretching vibration of FFA molecules [27], confirming the successful adsorption of FFA on the ZIS‐CDs surface. Under continuous illumination (0–60 min), the intensity of the O─H peak belonging to FFA decreased (Figure 3e), consistent with the consumption of FFA [26]. Simultaneously, two new peaks emerged at 1740 and 3500 cm^−1^, corresponding to the C═O and O─H stretching vibrations [38], respectively, which together confirm the continuous formation of FA. The conversion of FFA to FA was further verified by ^1^H and ^13^C nuclear magnetic resonance (NMR) spectroscopy (Figure S12). A characteristic peak near 1070 cm^−1^ (Figure 3e), whose intensity increased progressively with irradiation time, can be attributed to ·OOH [28]. Notably, the generated ·OOH can be further reduced by photogenerated electrons (e^−^) to yield the final product H_2_O_2_, providing direct spectroscopic evidence for the H_2_O_2_ formation pathway.

**FIGURE 4:**
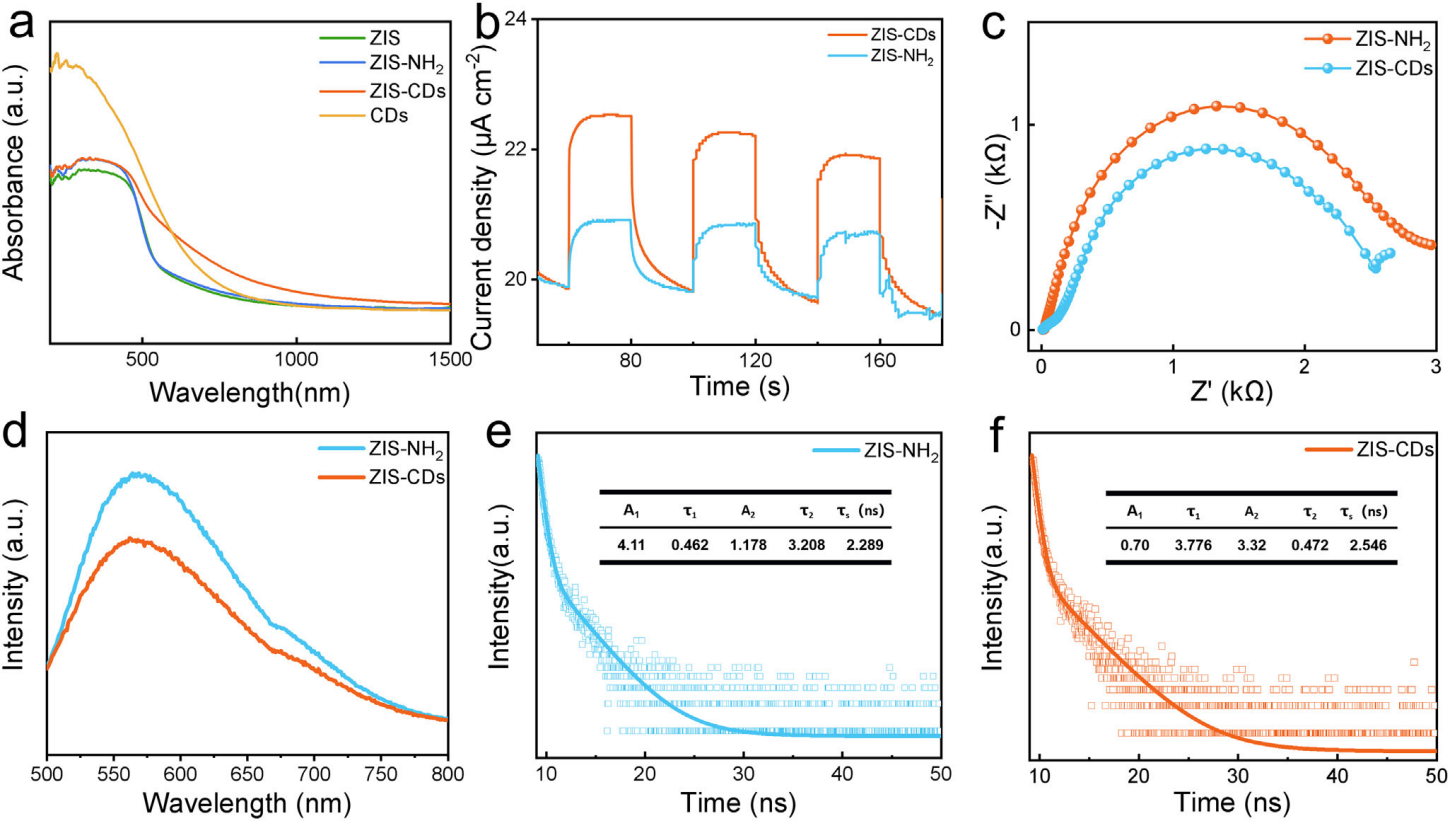
(a) UV–vis–NIR absorption spectra. (b) Photocurrent response curves of different samples and (c) EIS plots. (d) PL spectrum (e, f) time‐resolved photoluminescence (TRPL) decay curve of ZIS‐NH_2_ and ZIS‐CDs, with fitted lifetime parameters.

To further unravel the intrinsic mechanism governing the efficient photocatalytic H_2_O_2_ production and FFA conversion over the ZIS‐CDs heterojunction, this study systematically interrogated the separation and transfer dynamics of photogenerated charge carriers through a suite of complementary characterization techniques, including UV–vis–NIR absorption spectroscopy, transient photocurrent measurements, electrochemical impedance spectroscopy (EIS), photoluminescence (PL) spectroscopy, and band structure analysis. Figure 4a presents the UV–vis–NIR absorption spectra of the as‐prepared samples. It is evident that pristine ZIS exhibits predominant absorption in the ultraviolet (UV) region, whereas the ZIS‐CDs heterojunction displays remarkably enhanced and broadened light absorption spanning the 200–1500 nm range. This extended light‐harvesting capability facilitates the generation of a greater number of photogenerated electron‐hole pairs, thereby contributing to the improved photocatalytic performance [37]. Figure 4b depicts the transient photocurrent responses of ZIS‐NH_2_ and ZIS‐CDs upon intermittent light irradiation. Notably, the ZIS‐CDs heterojunction exhibits a substantially higher photocurrent density compared to pristine ZIS‐NH_2_. A higher photocurrent intensity is typically indicative of more efficient separation and faster migration of photoinduced charge carriers [51], which further supports the superior charge dynamics of the ZIS‐CDs heterojunction. Electrochemical impedance spectroscopy (EIS) measurements yielded Nyquist plots where the semicircle radius of ZIS‐NH_2_ was smaller than that of ZIS‐CDs [52]. This observation demonstrates that ZIS‐NH_2_ has enhanced charge transfer kinetics, which is one of the essential properties for high‐performance photocatalysts (Figure 4c). PL spectroscopy was employed to assess the recombination efficiency of photogenerated charge carriers. As shown in Figure 4d, the ZIS‐CDs sample exhibits a significantly lower PL intensity compared to the ZIS‐NH_2_ sample, which implies a substantially reduced recombination rate of photogenerated electron‐hole pairs. To quantitatively analyze the charge carrier lifetime, the PL decay curves were fitted using a double‐exponential model, and the average PL lifetime (*τ*_a_) was calculated in accordance with the following equation [2, 44]:

(1) $$\tau_{a}=\frac{A_{1}\tau_{1}^{2}+A_{2}\tau_{2}^{2}}{A_{1}\tau_{1}+A_{2}\tau_{2}}\;$$

where *A*_1_ and *A*_2_ correspond to the amplitudes, while *τ*_1_ and *τ*_2_ represent the short lifetime and long lifetime, respectively. The average exciton lifetime of ZIS‐CDs is longer than that of ZIS‐NH_2_ (Figure 4e,f), which indicates that efficient separation of photogenerated charge carriers is achieved in ZIS‐CDs through interfacial interactions, thereby suppressing charge recombination and enhancing photocatalytic efficiency [6, 8].

**FIGURE 5:**
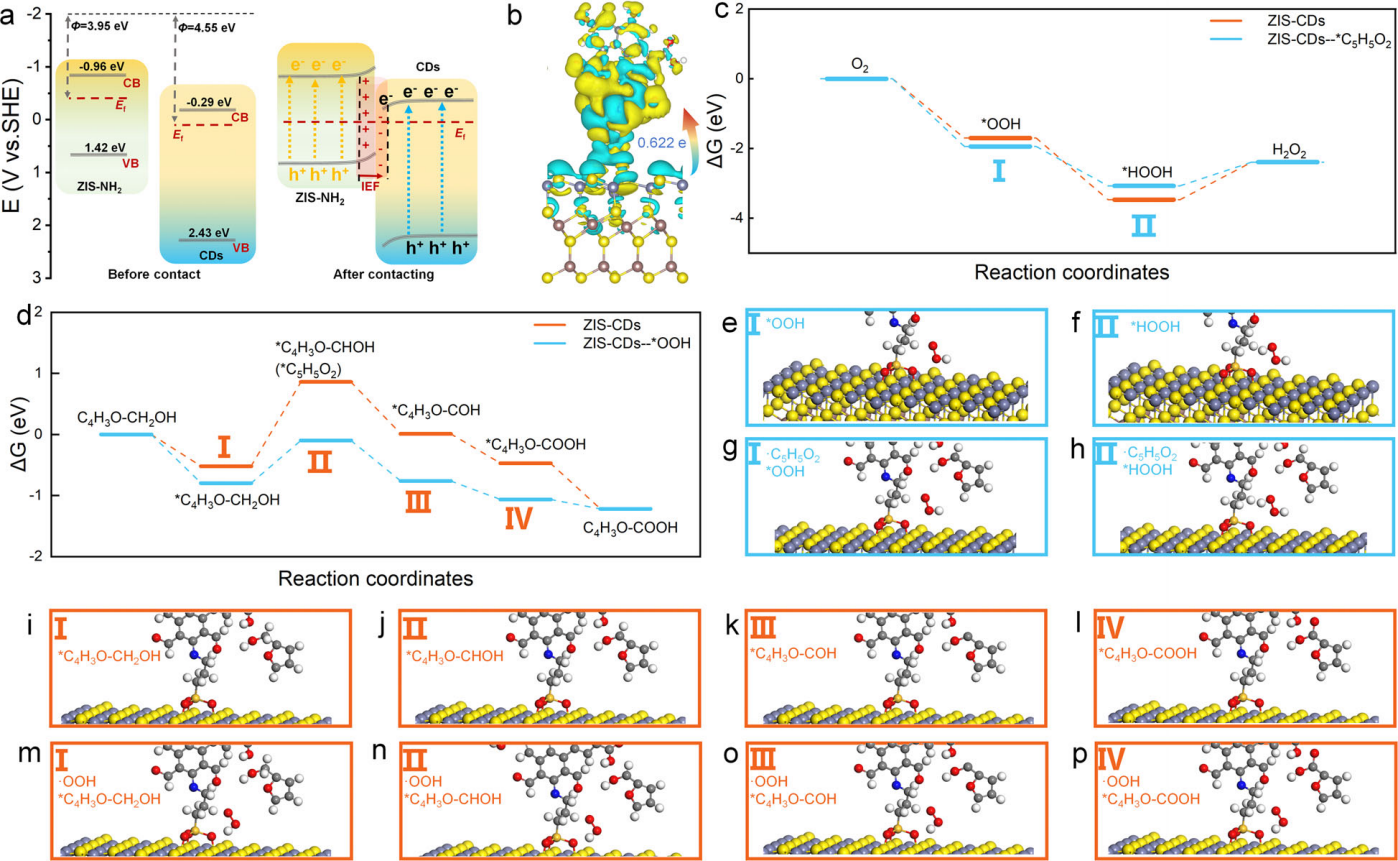
(a) Mechanism of Heterojunction Photocatalytic Hydrogen Peroxide Production by ZIS‐CDs. (b) Differential charge density map of ZIS‐CDs. (c)Free energy diagram of ZIS‐CDs for photocatalytic H_2_O_2_ synthesis by the 2e^−^ ORR path; The structure within the green rectangular box is the local structure of the O_2_ reduction intermediate adsorbed on the surface of ZIS‐CDs (e, f) without *C_5_H_5_O_2_, (g, h) with *C_5_H_5_O_2_. (d) Energy profile of the FFA oxidation process with O_2_ assistance; The structures within the orange rectangular frame are the local structures of the intermediates in the FFA oxidation process optimally adsorbed on the ZIS‐CDs surface (i–l) without *OOH, (m–p) with *OOH.

To elucidate the dynamic process of interfacial charge transfer, the band structures were systematically characterized via multiple techniques. First, the optical bandgaps (*E*_g_) of ZIS, ZIS‐NH_2_, ZIS‐CDs and CDs were calculated from UV–vis absorption spectra using the Tauc plots, yielding values of 2.33, 2.38, 2.44, and 2.72 eV (Figures S13 and S14), respectively. Subsequently, XPS valence band spectra were employed to directly determine the valence band maximum (*E*_VBM_) relative to the normal hydrogen electrode (NHE), with *E*_VBM_ values of 1.62 eV (ZIS), 1.42 eV (ZIS‐NH_2_),1.54 eV (ZIS‐CDs) and 2.43 eV (CDs). The conduction band minimum (*E*_CBM_) was further derived by the equation *E*_CBM_ = *E*_VBM_‐*E*_g_, resulting in values of −0.71 eV (ZIS), −0.96 eV (ZIS‐NH_2_), −0.9 eV (ZIS‐CDs), and −0.29 eV (CDs). Furthermore, ultraviolet photoelectron spectroscopy (UPS) measurements were performed with He I excitation (21.22 eV) [7]. As depicted in Figure S15, the cut‐off binding energies (*E*_cutoff_) of ZIS‐NH_2_ and CDs were determined as values of 17.27 and 16.67 eV, respectively. The work function (*Φ*) was calculated using the formula *Φ* = 21.22 eV—*E*_cutoff_, yielding *Φ* values of 3.95 eV for ZIS‐NH_2_ and 4.55 eV for CDs. Based on these results, the energy‐level alignment between ZIS‐NH_2_ and CDs is illustrated in Figure 5a. A staggered band alignment is observed, wherein both the conduction band (CB) and valence band (VB) of CDs are positioned at lower energy levels than those of ZIS‐NH_2_. Moreover, the Fermi level (*E*_f_) of ZIS‐NH_2_ is higher than that of CDs. Upon formation of intimate interfacial contact, electrons are spontaneously injected from ZIS‐NH_2_ with a relatively higher Fermi level toward CDs until their Fermi levels become aligned. This induces positive charging and upward band bending on the ZIS side, alongside negative charging and downward band bending on the CDs side, thus establishing a built‐in electric field directed from ZIS to CDs. To further verify the interfacial charge transfer behavior, density functional theory (DFT) simulations were carried out to calculate the differential charge density of the ZIS‐CDs heterojunction (Figure 5b). In the differential charge density, blue and yellow regions correspond to electron depletion and accumulation, respectively. Notably, electron accumulation is observed on the CDs side, whereas electron depletion occurs on the ZIS side, accompanying a net charge transfer of 0.622 e from ZIS to CDs. These results are highly consistent with the band structure analysis as well as XPS measurements, collectively confirming the directional charge transfer at the heterogeneous interface.

Based on the above analyses, ZIS and CDs are interconnected via a Schiff‐base reaction between the amino groups of APTES and the aldehyde groups on CDs. Upon light excitation, the built‐in electric field across the as‐formed heterojunction significantly facilitates the separation of photogenerated electron–hole pairs. Furthermore, photogenerated electrons rapidly transfer from the conduction band (CB) of CDs to the valence band (VB) of ZIS through the covalent‐bond linkage of APTES. This unique charge transfer pathway thus retains highly reductive electrons in the CB of ZIS and highly oxidative holes in the VB of CDs. Specifically, the photogenerated electrons migrate to the ZIS surface to participate in the ORR for H_2_O_2_ generation, while the photogenerated holes migrate to the CDs surface to drive FFA conversion. The overall coupled reaction can be summarized as follows:

(2) $$O_{2}+e^{-}\to \cdot {O_{2}}^{-}$$

(3) $$\cdot {O_{2}}^{-}+H^{+}\to \cdot \mathrm{OOH}$$

(4) $$\cdot \mathrm{OOH}+H^{+}+e^{-}\to *\mathrm{HOOH}$$

(5) $$*\mathrm{HOOH}\to H_{2}O_{2}$$

(6) $$C_{4}H_{3}O-CH_{2}-\mathrm{OH}+h^{+}\to C_{4}H_{3}O-\cdot \mathrm{CH}-\mathrm{OH}+H^{+}$$

(7) $$C_{4}H_{3}O-\cdot \mathrm{CH}-\mathrm{OH}+h^{+}\to C_{4}H_{3}O-\cdot C\cdot -\mathrm{OH}+H^{+}$$

(8) $$2C_{4}H_{3}O-\cdot C\cdot -\mathrm{OH}+O_{2}\to 2C_{4}H_{3}O-\mathrm{COOH}$$

As illustrated in the reaction scheme, hydrogen ions generated during FFA conversion exactly act as the proton source for the oxygen reduction reaction (ORR), while the reactive intermediate ·O_2_^−^ involved in H_2_O_2_ generation also participates in FA formation. The coupling of these two reactions is the key to achieving highly efficient synergy between photocatalytic H_2_O_2_ generation and FFA photooxidation. Subsequently, we investigated the kinetic process of the coupled reaction via density functional theory (DFT) calculations. As shown in Figure 5c,e–h, in the presence of the ·C_5_H_5_O_2_ intermediate (corresponding to the C_4_H_3_O‐·CH‐OH species), the energy barrier for the rate‐determining step (RDS) in the ZIS‐CDs catalytic system is significantly lower than that in the system without this intermediate. This result directly confirms that the C_4_H_3_O‐·CH‐OH intermediate effectively promotes the reduction of O_2_ to H_2_O_2_. Detailed mechanistic studies revealed that under light excitation, photogenerated holes from the ZIS‐CDs heterojunction first activate FFA molecules (C_4_H_3_O‐CH_2_‐OH) adsorbed at the C═N‐linked active sites formed via Schiff base coupling, cleaving the α─C─H bond to generate the C_4_H_3_O‐·CH‐OH intermediate. The hydrogen proton is then captured by ·O_2_^−^, detaches from the α‐carbon, and undergoes reaction to form ·OOH [4]. Subsequently, photogenerated holes act again on the α─C─H bond of C_4_H_3_O‐·CH‐OH producing C_4_H_3_O‐·C·‐OH with a triplet carbene structure. Simultaneously, the hydrogen proton is captured by ·OOH, departs from the α‐carbon, and reacts to form *HOOH, which then desorbs to afford free H_2_O_2_. Two molecules of C_4_H_3_O‐·C·‐OH react with abundant O_2_ in the system to generate two molecules of FA, which ultimately desorb from the ZIS‐CDs catalyst surface, completing the entire reaction cycle. To validate this proposed reaction pathway, DFT calculations were performed to map the complete reaction route for ZIS‐CDs‐catalyzed FFA oxidation (Figure 5d,i‐p). The results indicate that the RDS is the deprotonation of the α─C─H bond, with a reaction barrier of 1.380 eV, while all other steps proceed spontaneously with a decrease in free energy. Importantly, in the presence of the *OOH intermediate, the barrier for the RDS is significantly reduced to 0.699 eV (Figure 5d), demonstrating that the *OOH intermediate markedly accelerates the conversion of FFA to FA. On the whole, the synergistic interplay between the ·C_5_H_5_O_2_ and ·OOH intermediates significantly enhance the redox activity of the system. This finding offers direct theoretical evidence for the efficient separation and utilization of photogenerated carriers, as well as the optimized supply of hydrogen ions, in the ZIS‐CDs‐catalyzed acetonitrile‐furfuryl alcohol system.

## 3 Conclusions

In summary, we have successfully developed a ZIS‐CDs heterogeneous photocatalyst for efficient H_2_O_2_ production coupled with photosynthesis of FA. The ZIS nanobelts and CDs are covalently linked via 3‐aminopropyltriethoxysilane through a Schiff‐base reaction between the amino groups of ZIS‐NH_2_ and carbonyl groups of CDs. This robust covalent connection, together with the built‐in electric field in the interface of the ZIS‐CDs heterojunction, constructs an efficient electron transfer channel, effectively promoting the separation, directional migration, and respective reactions of photogenerated electron‐hole. In contrast to conventional aqueous photocatalytic H_2_O_2_ production systems, this work couples FFA oxidation with H_2_O_2_ generation in an ACN‐based medium. FFA serves as a sacrificial electron donor, oxidation substrate, and proton source, enabling efficient H_2_O_2_ formation while being selectively converted into FA. The system achieves nearly 100% selectivity toward FA and 98% FFA conversion. Meanwhile, the photoinduced electron and reacts with the hydrogen proton and O_2_ to produce H_2_O_2_, reaching a maximum rate of 24 mmol·g^−1^·h^−1^, the AQY value of 14.5% at 400 nm and the SCC efficiency of 1.78%. Theoretical calculations confirm that ZIS‐CDs can effectively enhance substrate activation and the dehydrogenation process of FFA, thereby accelerating the reaction kinetics of both H_2_O_2_ production and FFA photooxidation and significantly improving the overall reaction efficiency. Overall, this work provides a feasible solution to the proton supply challenge in efficient H_2_O_2_ synthesis, offers new insights for the development of synergistic conversion strategies.

## Author Contributions

P. B. and N. L. contributed equally to this work. P. B.: methodology, investigation, data curation, writing – original draft; N. L.: conceptualization, software, funding acquisition, writing – original draft; Q. Z.: investigation, data curation; B. L., X. F. and L. L.: formal analysis, visualization; J. L.: data curation, formal analysis; W. J.: investigation, writing – review and editing; S. H.: supervision, project administration, writing – review and editing; H. Z.: conceptualization, funding acquisition, writing – review and editing.

## Conflicts of Interest

The authors declare no conflicts of interest.

## Supporting information




**Supporting File**: advs76085‐sup‐0001‐SuppMat.docx.

## Data Availability

The data that support the findings of this study are available from the corresponding author upon reasonable request.

## References

[1] Y. Yi , L. Wang , G. Li , and H. Guo , “A Review on Research Progress in the Direct Synthesis of Hydrogen Peroxide From Hydrogen and Oxygen: Noble‐Metal Catalytic Method, Fuel‐Cell Method and Plasma Method,” Catalysis Science & Technology 6 (2016): 1593–1610, 10.1039/c5cy01567g.

[2] Z. Li , J. Zi , X. Luan , et al., “Localized Surface Plasmon Resonance Promotes Metal–Organic Framework‐Based Photocatalytic Hydrogen Evolution,” Advanced Functional Materials 33 (2023): 2303069, 10.1002/adfm.202303069.

[3] X. Zhang , H. Su , P. Cui , et al., “Developing Ni Single‐Atom Sites in Carbon Nitride for Efficient Photocatalytic H_2_O_2_ Production,” Nature Communications 14 (2023): 7115, 10.1038/s41467-023-42887-y. PMC10628073 37932292

[4] A. Chakraborty , A. Alam , U. Pal , et al., “Enhancing Photocatalytic Hydrogen Peroxide Generation by Tuning Hydrazone Linkage Density in Covalent Organic Frameworks,” Nature Communications 16 (2025): 503, 10.1038/s41467-025-55894-y. PMC11711387 39779748

[5] Y. Isaka , Y. Kawase , Y. Kuwahara , K. Mori , and H. Yamashita , “Two‐Phase System Utilizing Hydrophobic Metal–Organic Frameworks (MOFs) for Photocatalytic Synthesis of Hydrogen Peroxide,” Angewandte Chemie International Edition 58 (2019): 5402–5406, 10.1002/anie.201901961. 30793452

[6] Q. Zhu , J. Su , G. Lin , et al., “Surface Indium Vacancies Promote Photocatalytic H_2_O_2_ Production Over In_2_S_3_ ,” Nature Communications 16 (2025): 10501, 10.1038/s41467-025-65538-w. PMC12647849 41290601

[7] X. Zhang , D. Gao , B. Zhu , B. Cheng , J. Yu , and H. Yu , “Enhancing Photocatalytic H_2_O_2_ Production With Au Co‐Catalysts Through Electronic Structure Modification,” Nature Communications 15 (2024): 3212, 10.1038/s41467-024-47624-7. PMC11016070 38615063

[8] P. Liu , T. Liang , Y. Li , et al., “Photocatalytic H_2_O_2_ Production Over Boron‐Doped G‐C_3_N_4_ Containing Coordinatively Unsaturated FeOOH Sites and CoO_x_ Clusters,” Nature Communications 15 (2024): 9224, 10.1038/s41467-024-53482-0. PMC11511943 39455557

[9] H. Tan , P. Zhou , M. Liu , et al., “Al–N_3_ Bridge Site Enabling Interlayer Charge Transfer Boosts the Direct Photosynthesis of Hydrogen Peroxide From Water and Air,” Journal of the American Chemical Society 146 (2024): 31950–31960, 10.1021/jacs.4c11471. 39500575

[10] C. W. Bai , L. L. Liu , J. J. Chen , et al., “Circumventing Bottlenecks in H_2_O_2_ Photosynthesis Over Carbon Nitride With Iodine Redox Chemistry and Electric Field Effects,” Nature Communications 15 (2024): 4718, 10.1038/s41467-024-49046-x. PMC11535034 38830881

[11] K. Meng , J. Zhang , B. Cheng , et al., “Plasmonic Near‐Infrared‐Response S‐Scheme ZnO/CuInS_2_ Photocatalyst for H_2_O_2_ Production Coupled With Glycerin Oxidation,” Advanced Materials 36 (2024): 2406460, 10.1038/s44160-025-00880-x. 38837488

[12] M. Gu , D. Y. Lee , J. Mun , et al., “Solar‐to‐Hydrogen Peroxide Conversion of Photocatalytic Carbon Dots With Anthraquinone: Unveiling the Dual Role of Surface Functionalities,” Applied Catalysis B: Environmental 312 (2022): 121379, 10.1016/j.apcatb.2022.121379.

[13] H. Guo , S. Wang , X. Chen , et al., “Engineering a Covalent Organic Framework‐Based Type‐II Heterojunction for Enhanced Photocatalytic H_2_O_2_ Synthesis,” Nature Synthesis 4 (2025): 1610–1620, 10.1002/adma.202406460.

[14] C. Shu , X. Yang , L. Liu , et al., “Mixed‐Linker Strategy for the Construction of Sulfone‐Containing D–A–A Covalent Organic Frameworks for Efficient Photocatalytic Hydrogen Peroxide Production,” Angewandte Chemie International Edition 63 (2024): 202403926, 10.1002/anie.202403926. 38414401

[15] J. Hu , B. Li , X. Li , et al., “Lattice Match‐Enabled Covalent Heterointerfaces With Built‐In Electric Field for Efficient Hydrogen Peroxide Photosynthesis,” Advanced Materials 36 (2024): 2412070, 10.1002/adma.202412070. 39428842

[16] X. Ruan , S. Zhao , M. Xu , et al., “Iso‐Elemental ZnIn_2_S_4_/Zn_3_In_2_S_6_ Heterojunction With Low Contact Energy Barrier Boosts Artificial Photosynthesis of Hydrogen Peroxide,” Advanced Energy Materials 14 (2024): 2401744, 10.1002/aenm.

[17] J. Qiu , K. Meng , Y. Zhang , et al., “COF/In_2_S_3_ S‐Scheme Photocatalyst With Enhanced Light Absorption and H_2_O_2_‐Production Activity and fs‐TA Investigation,” Advanced Materials 36 (2024): 2400288, 10.1002/adma.202400288. 38411357

[18] K. Zhang , M. Dan , J. Yang , et al., “Surface Energy Mediated Sulfur Vacancy of ZnIn_2_S_4_ Atomic Layers for Photocatalytic H_2_O_2_ Production,” Advanced Functional Materials 33 (2023): 2302964, 10.1002/adfm.202302964.

[19] J. Zhou , Y. Mu , M. Qiao , et al., “Unlocking One‐Step Two‐Electron Oxygen Reduction via Metalloid Boron‐Modified Zn_3_in_2_S_6_ for Efficient H_2_O_2_ Photosynthesis,” Angewandte Chemie International Edition 64 (2025): 202506963, 10.1002/anie.202506963. 40317879

[20] M. Li , C. Ling , L. Zhao , L. Hu , H. Li , and L. Zhang , “Surface Fe^IV^ = O Induced Highly Selective Phenol Polymerization via Proton‐Coupled Electron Transfer,” Journal of the American Chemical Society 147 (2025): 31165–31174, 10.1021/jacs.5c09972. 40801075

[21] W. Yang , M. Li , B. Zhang , et al., “Interfacial Microenvironment Modulation Boosts Efficient Hydrogen Evolution Reaction in Neutral and Alkaline,” Advanced Functional Materials 33 (2023): 2304852, 10.1002/adfm.202304852.

[22] J. Huang , R. Wang , H. Sheng , et al., “Isotope‐Dependent Tafel Analysis Probes Proton Transfer Kinetics during Electrocatalytic Water Splitting,” Nature Chemistry 18 (2026): 669–676, 10.1038/s41557-025-01934-5. PMC13061611 40925945

[23] C. Sun , Y. Han , H. Guo , et al., “Proton Reservoir in Covalent Organic Framework Compensating Oxygen Reduction Reaction Enhances Hydrogen Peroxide Photosynthesis,” Advanced Materials 37 (2025): 2502990, 10.1002/adma.202502990. 40159893

[24] Q. Zhu , L. Shi , Z. Li , G. Li , and X. Xu , “Protonation of an Imine‐Linked Covalent Organic Framework for Efficient H_2_O_2_ Photosynthesis Under Visible Light up to 700 nm,” Angewandte Chemie International Edition 63 (2024): 202408041, 10.1002/anie.202408041. 38738797

[25] L. Li , X. Lv , Y. Xue , H. Shao , G. Zheng , and Q. Han , “Custom‐Design of Strong Electron/Proton Extractor on COFs for Efficient Photocatalytic H_2_O_2_ Production,” Angewandte Chemie International Edition 63 (2024): 202320218, 10.1002/anie.202320218. 38353181

[26] Y. Yang , J. Liu , M. Gu , B. Cheng , L. Wang , and J. Yu , “Bifunctional TiO_2_/COF S‐scheme Photocatalyst With Enhanced H_2_O_2_ Production and Furoic Acid Synthesis Mechanism,” Applied Catalysis B: Environment and Energy 333 (2023): 122780, 10.1016/j.apcatb.2023.122780.

[27] J. Liu , C. Tuo , W. Xiao , et al., “Constructing Donor‐Acceptor Covalent Organic Frameworks for Highly Efficient H_2_O_2_ Photosynthesis Coupled With Oxidative Organic Transformations,” Angewandte Chemie International Edition 64 (2025): 202416240, 10.1002/anie.202416240. 39299929

[28] L. Xu , K. S. Yeung , L. Li , et al., “Production of H_2_O_2_ via Energy Transfer Photocatalysis by Coupling With Furfuryl Alcohol Conversion Over an Amide‐Functionalized Heptazine Framework,” Angewandte Chemie International Edition 64 (2025): 202504635, 10.1002/anie.202504635. 40302651

[29] S. Li , R. Ma , C. Tu , et al., “Programmed Charge Transfer in Conjugated Polymers With Pendant Benzothiadiazole Acceptor for Simultaneous Photocatalytic H_2_O_2_ Production and Organic Synthesis,” Angewandte Chemie International Edition 64 (2025): 202421040, 10.1002/anie.202421040. 39539214

[30] J. Chang , J. Shi , Q. Li , et al., “Regulation of Redox Molecular Junctions in Covalent Organic Frameworks for H_2_O_2_ Photosynthesis Coupled With Biomass Valorization,” Angewandte Chemie International Edition 62 (2023): 202303606, 10.1002/anie.202303606. 37277319

[31] Q. Xue , H. Li , P. Jin , X. Zhou , and F. Wang , “Singlet‐Oxygen‐Driven Cooperative Photocatalytic Coupling of Biomass Valorization and Hydrogen Peroxide Production Using Covalent Organic Frameworks,” Angewandte Chemie International Edition 64 (2025): 202423368, 10.1002/anie.202423368. 40035701

[32] X. Han , Y. Wang , G. Liu , M. Wang , C. Guo , and J. Shen , “A Sustainable and Low‐Cost Route to 2,5‐Furandicarboxylic Acid by Carboxylation of Biomass‐Based Furoic Acid and CO_2_ ,” Journal of CO2 Utilization 75 (2023): 102572, 10.1016/j.jcou.2023.102572.

[33] K. Wang , C. Liang , S. Fu , et al., “Molecularly Tailored Carbon Quantum Dots Enable Enhanced C‐C Coupling in CO_2_ Electroreduction,” Advanced Functional Materials 36 (2025): 202530561, 10.1002/adfm.202530561.

[34] J. Hu , T. Yang , J. Chen , X. Yang , J. Qu , and Y. Cai , “Efficient Solar‐Driven H_2_O_2_ Synthesis In‐Situ and Sustainable Activation to Purify Water via Cascade Reaction on ZnIn_2_S_4_‐Based Heterojunction,” Chemical Engineering Journal 430 (2022): 133039, 10.1016/j.cej.2021.133039.

[35] Y. Shao , J. Hu , T. Yang , et al., “Significantly Enhanced Photocatalytic In‐Situ H_2_O_2_ Production and Consumption Activities for Efficient Sterilization by ZnIn_2_S_4_/G‐C_3_N_4_ Heterojunction,” Carbon 190 (2022): 337–347, 10.1016/j.carbon.2022.01.019.

[36] Y. Tang , J. Qiu , D. Dai , et al., “ZnIn_2_S_4_/MIL‐53‐NH_2_ Composite Photocatalysts for H_2_O_2_ Production: Synergistic Effect of Sulfur Vacancy and Heterostructure,” Separation and Purification Technology 354 (2025): 129350, 10.1016/j.seppur.2024.129350.

[37] N. Li , J. Ma , W. Wang , et al., “Dual S‐Scheme MoS_2_/ZnIn_2_S_4_/Graphene Quantum Dots Ternary Heterojunctions for Highly Efficient Photocatalytic Hydrogen Evolution,” Journal of Colloid and Interface Science 676 (2024): 496–505, 10.1016/j.jcis.2024.07.144. 39047377

[38] T. Xiao , L. Wang , K. Li , et al., “Donor‐Acceptor‐Donor Organic Small Molecules as Hole Transfer Vehicle Covalently Coupled Znln_2_S_4_ Nanosheets for Efficient Photocatalytic Hydrogen Evolution,” Advanced Functional Materials 35 (2025): 2412644, 10.1002/adfm.202412644.

[39] H. Oh and J. Kim , “Fabrication of Polymethyl Methacrylate Composites With Silanized Boron Nitride by In‐Situ Polymerization for High Thermal Conductivity,” Composites Science and Technology 172 (2019): 153–162, 10.1016/j.compscitech.2019.01.021.

[40] Z. Liu , J. Li , and X. Liu , “Novel Functionalized BN Nanosheets/Epoxy Composites With Advanced Thermal Conductivity and Mechanical Properties,” ACS Applied Materials & Interfaces 12 (2020): 6503–6515, 10.1021/acsami.9b21467. 31933354

[41] M. Xu , M. Lu , G. Qin , et al., “Piezo‐Photocatalytic Synergy in BiFeO_3_ @COF Z‐Scheme Heterostructures for High‐Efficiency Overall Water Splitting,” Angewandte Chemie International Edition 61 (2022): 202210700, 10.1002/ange.202210700. 36098495

[42] L. Xie , C. Liang , H. Guo , et al., “N‐B‐O‐Crosslinker‐Induced Mechanochemical Conversion of Carbon Phase Unlocks Efficient Hydrogen Peroxide Electrosynthesis,” Chinese Chemical Letters 37 (2026): 111934, 10.1016/j.cclet.2025.111934.

[43] X. Li , R. Hu , Y. Liu , et al., “Co‐Construction of Oxygen Doping and Van Der Walls Heterojunction in O‐CB/ZnIn_2_S_4_ Promoting Photocatalytic Production and Activation of H_2_O_2_ for the Degradation of Antibiotics,” Journal of Hazardous Materials 459 (2023): 132187, 10.1016/j.jhazmat.2023.132187. 37541119

[44] Y. Xu , J. Liao , L. Zhang , Z. Sun , and C. Ge , “Dual Sulfur Defect Engineering of Z‐Scheme Heterojunction on Ag‐CdS_1‐x_@ZnIn_2_S_4‐x_ Hollow Core‐Shell for Ultra‐Efficient Selective Photocatalytic H_2_O_2_ Production,” Journal of Colloid and Interface Science 647 (2023): 446–455, 10.1016/j.jcis.2023.05.140. 37271089

[45] C. Wang , H. Li , F. Shen , et al., “A Strong Metal‐Support Interaction Strategy for Enhanced Proton‐Coupled Electron Transfer and Promoted Photocatalytic H_2_O_2_ Production in Pure Water,” Applied Catalysis B: Environment and Energy 361 (2025): 124672, 10.1016/j.apcatb.2024.124672.

[46] Z. Zhao , R. Sun , Y. Wang , et al., “Fused‐Ring Acceptor‐π‐Acceptor Architecture Enables Near‐Infrared‐Absorbing Mesoporous Covalent Organic Frameworks for Enhanced H_2_O_2_ Photosynthesis,” Angewandte Chemie International Edition 129 (2026): e5267333, 10.1002/anie.202507333. 41858167

[47] H. Zhang , R. Ma , K. Chi , Y. Liu , and Y. Zhao , “Hydrogen Radical Mediated Concerted Electron−Proton Transfer in 1D Sulfone‐Based Covalent Organic Framework for Boosting Photosynthesis of H_2_O_2_ ,” Angewandte Chemie International Edition 64 (2025): 202516657, 10.1002/anie.202516657. 40923463

[48] M. Gu , Y. Yang , L. Zhang , B. Zhu , G. Liang , and J. Yu , “Efficient Sacrificial‐Agent‐Free Solar H_2_O_2_ Production Over All‐Inorganic S‐Scheme Composites,” Applied Catalysis B: Environmental 324 (2023): 122227, 10.1016/j.apcatb.2022.122227.

[49] S. Y. Cheng , Y. Cheng , C. X. Li , et al., “Rare‐Earth Metal–Organic Framework/CdS Heterostructures for Highly Efficient Photocatalytic Hydrogen Evolution,” Advanced Synthesis & Catalysis 368 (2026): 70357, 10.1002/adsc.70357.

[50] C. Zhu , Y. Yao , Q. Fang , S. Song , B. Chen , and Y. Shen , “Unveiling the Dynamic Evolution of Single‐Atom Co Sites in Covalent Triazine Frameworks for Enhanced H_2_O_2_ Photosynthesis,” ACS Catalysis 14 (2024): 2847–2858, 10.1021/acscatal.3c04439.

[51] J. Liu , M. Zhu , W. Yuan , et al., “Lamellar WO_3_/AgI S‐Scheme Heterojunction for Superior Visible Light Driven Photocatalytic Degradation of Ciprofloxacin,” Chemical Engineering Science 285 (2024): 119639, 10.1016/j.ces.2023.119639.

[52] Y. Huang , S. Jian , Z. Guo , et al., “Guest‐Enhanced Charge Separation in Porphyrin‐Based Double‐Cavity Metallacages for Visible‐Light‐Driven H_2_O_2_ Production,” Angewandte Chemie International Edition 65 (2026): 9964829, 10.1002/anie.202509482. 41841188

