# Supplementary material for: Furfuryl Alcohol‐Driven Proton Supply Enables Efficient Photocatalytic H2O2 Production Beyond Water‐Based Systems
Source: Adv Sci (Weinh). 2026 Jun 11;13(50):e76085. doi: 10.1002/advs.76085 (PMC13336625; doi:10.1002/advs.76085)
Supplement: Supplementary file 1 [file ADVS-13-e76085-s001.docx]

**Supporting Information**

**Furfuryl Alcohol–Driven Proton Supply Enables Efficient Photocatalytic H_2_O_2_ Production beyond Water-Based Systems**

Pengfei Bai^a,1^, Ning Li^a,1,*^, Quan Zhou^a^, Bin Liu^a^, Xiangqian Fan^a^, Lei Liu^a^, Jingkai Lin^b^, Weijie Ren ^c,*^, Shengliang Hu^a,*^, Huayang Zhang^b,*^

^a^School of Energy and Power Engineering & State Key Laboratory of Coal and CBM Co-Mining, North University of China, Taiyuan 030051, China.

^b^School of Chemical Engineering, Adelaide University, Adelaide, SA 5005, Australia.

^c^Shanxi Center of Technology Innovation for Light Manipulations and Applications, School of Applied Science, Taiyuan University of Science and Technology, Taiyuan 030024, China

Correspondences: lnlong2834@yeah.net; hsliang@yeah.net; renweijie@163.com; huayang.zhang@adelaide.edu.au

^1^Pengfei Bai and Ning Li contributed equally to this work.

**Experimental Section**

**Materials:** Zinc sulfate heptahydrate (ZnSO_4_·7H_2_O, 99.995% metal basis), Indium chloride (InCl_3_, 99.99% metal basis), thioacetamide (CH_3_CSNH_2_, TAA, ≥98%), Acetonitrile（CH_3_CN, HPLC, 99.9%, Furfuryl alcohol (C_5_H_6_O_2_, >98%) and Pyridine (C_5_H_5_N, 99.5%) were purchased from Aladdin Biochemical Technology Co., Ltd.

**Synthesis of ZIS, ZIS-NH_2_, and ZIS-CDs samples:** Pure ZIS was synthesized via a one-step hydrothermal method. Specifically, 1 mmol of InCl_3_, 0.5 mmol of ZnSO_4_·7H_2_O, and 4 mmol of thioacetamide (TAA) were sequentially dissolved in 24 mL of pyridine. The resulting solution was stirred for 60 minutes and then transferred into a 50 mL stainless-steel autoclave lined with polytetrafluoroethylene (PTFE). The autoclave was heated to 200 °C and maintained at this temperature for 16 hours. After natural cooling, the product was collected by centrifugation, repeatedly washed with absolute ethanol, and dried in an oven at 60 °C for 4 hours. The dried ZIS powder was dispersed in 50 mL of absolute ethanol, followed by the addition of 1 mL of 3-aminopropyltriethoxysilane (APTES) [S1]. The mixture was stirred (600 rpm) through oil bath at 60 °C for 3 hours. After natural cooling, the product was collected by centrifugation, repeatedly washed with absolute ethanol and dried. Subsequently, ZIS-NH_2_ powder was dispersed in 45 mL of absolute ethanol, and 5 mL of CDs solution was added. Then the parameters of the oil bath were set as 400 rpm, 80 °C and 8 hours. After natural cooling, the ZIS-CDs product was collected by centrifugation, repeatedly washed with absolute ethanol and dried.

**Characterization:** The morphology and microstructure were characterized using a Sigma 300 scanning electron microscope (SEM) equipped with an Energy Dispersive Spectrometer (EDS), Talos F200X G2 transmission electron microscope (TEM). Crystal structure and phase information were analyzed by X-ray diffraction (XRD) measurements (Smart Lab SE). The chemical composition and electronic states were investigated using X-ray photoelectron spectrometer (XPS, ESCALAB 250XI), with the C 1s peak serving as the reference for binding energy calibration. Ultraviolet photoelectron spectroscopy (UPS) measurements were conducted on an ESCALAB 250 Xi system to determine the cutoff energy values, with a photon emission energy of 21.22 eV through He I excitation. Brunauer-Emmett-Teller (BET) measurements were performed to determine the specific surface area and pore structure characteristics of the samples. Steady-state photoluminescence (PL) spectra and time-resolved photoluminescence (TRPL) decay curves were measured using a FLS1000 steady-state/transient fluorescence spectrometer (Edinburgh Instruments, UK). In-situ diffuse reflectance infrared Fourier transform spectroscopy (DRIFTS) was utilized to explore the surface-adsorbed species and reaction intermediates involved in the catalytic reaction.

**Photocatalytic production of H_2_O_2_****:** The photocatalytic H_2_O_2_ generation was conducted in a custom-built recirculation reactor. Typically, 5.0 milligrams of catalyst is dispersed in 30 min of 10% furfuryl alcohol acetonitrile solution, and before illumination, the system is flushed with high-purity O_2_ for 30 minutes in the dark. A 300 W xenon lamp (100 mW cm^−2^ intensity, 4.0 cm^2^ illumination area) was used as the light source. The temperature maintained at 15.0 ± 0.5 °C using a recirculating water cooling system. H_2_O_2_ concentration was quantified by iodometric titration using a UV−Vis spectrophotometer. At 30-minute intervals, 1 mL of the reaction solution was collected, filtered, and mixed with 0.5 mL of 0.4 M KI and 0.5 mL of 0.1 M potassium hydrogen phthalate buffer. After 30 minutes of reaction in the dark, absorbance at 350 nm was measured to calculate H_2_O_2_ yield.

H_2_O_2_ + 3I^−^ + 2H^+^ → I_3_^−^ + 2H_2_O	(1)

I_3_^−^ exhibits a strong absorption at 350 nm, allowing the concentration of H_2_O_2_ to be determined by recording UV−Vis absorption spectra.

**Statistical Analysis:** Statistical analysis was performed using Origin software (version 2018). All independent experiments were repeated in triplicate (n = 3), and the results are presented as mean ± standard deviation (SD). Statistical significance levels (*p < 0.05, **p < 0.01, ***p < 0.001) were defined through one-way analysis of variance (ANOVA).

**Apparent quantum yield (AQY) and Solar-to-chemical conversion (SCC) efficiencies:** To determine the apparent quantum yield (AQY), 400, 420,500,600 and 700 nm LED lamps were employed as light sources, with the illumination area fixed at 0.81 cm^2^ and irradiation duration set to 1 hour. Light intensity at each wavelength was measured using an optical power meter for accurate AQY calculations. Apparent quantum yield (AQY) can be calculated using the following formula.

$AQY(\%)=\frac{2\times N_{H_{2}O_{2}}}{N_{photons}}\times100\%$	(2)

Where $N_{H_{2}O_{2}}$ is the number of H_2_O_2_ molecules produced and *N*_photons_ is the number of incident photons.

A 300 W xenon lamp was employed to simulate solar light irradiation at 100 mW cm^−2^. 20.0 mg of the catalyst was used in the photocatalytic reaction.

$SCC(\%)=\frac{{\Delta G}_{H_{2}O_{2}}\times N_{H_{2}O_{2}}}{I\times S\times T}\times100\%$	(3)

Where ${\Delta G}_{H_{2}O_{2}}$= 117 KJ mol^−1^. In addition, *I*, *S*, and *T* represent the light intensity (100 mW cm^−2^), irradiation area (4.0 cm^2^), and irradiation time (1 h), respectively.

**Photoelectrochemical and electrochemical measurements:** To evaluate the photoelectrochemical performance of materials, a three-electrode testing system using a Biologic SP-200 electrochemical workstation was employed. The working electrode, the counter electrode and the reference electrode were catalyst-coated ITO conductive glass (2 cm × 3 cm), platinum plate (1 cm^2^) and saturated Ag/AgCl (containing saturated potassium chloride electrolyte), respectively. The working electrode preparation involved: ultrasonically dispersing 10 mg catalyst in 100 μL of anhydrous ethanol, uniformly coating the suspension onto ITO substrate, followed by vacuum drying at 60 °C for 1 hour. The electrolyte was 0.5 M Na_2_SO_4_ solution. The photocurrent response was tested under 0.4 V bias voltage and 300 W Xenon lamp, and the light source automatically turned on/off every 20 seconds. EIS test was recorded in the frequency range from 0.01 Hz to 105 Hz with 0.005 V of the amplitude. Rotating ring disk electrode (RRDE) analysis.H_2_O_2_ selectivity was evaluated using a rotating ring-disk electrode (RRDE) coupled with a CHI-760E electrochemical workstation. The three-electrode system consisted of a glassy carbon disk with catalyst and platinum ring (0.1963 cm^2^) as the working electrode, a Ag/AgCl reference electrode, and a Pt/C counter electrode. The electrolyte was an O_2_-saturated 0.1 M Na_2_SO_4_ solution (pH = 6.8). The RRDE rotation speed was maintained at 1600 rpm, with a potential scan from −0.87 V to 0.39 V *vs*. Ag/AgCl. The working electrode was prepared as follows: Initially, 10.0 mg of catalyst was dispersed in 1 mL of ethanol with 10 μL of Nafion by ultrasonication for 30 minutes. Then, 20 μL of the suspension was drop-casted onto the RRDE and dried. The H_2_O_2_ selectivity was calculated according to the following formula:

$H_{2}O_{2} Selectivity (\%)=2\times\frac{I_{r}/N}{I_{d}+I_{r}/N}$×100%	(4)

*I*_r_ is the ring current. *I*_d_ is the disc current. *N* is the collection efficiency (N = 0.35).

Calculation: Density Functional Theory (DFT) calculations were performed by using the CP2K package. by using the CP2K package mixed Gaussian and plane-wave schem[S2]and the Quickstep module[S3]. The Perdew-Burke-Ernzerhof (PBE) exchange correlation functional[S4], Goedecker-Teter-Hutter (GTH) pseudopotential, DZVP-MOLOPT-SR-GTH / TZVP-MOLOPT-SR-GTH / TZVP-MOLOPT-GTH basis sets were used to describe the system[S5]. A plane-wave energy cut-off and relative cut-off of 400 Ry and 55 Ry have been employed, respectively. The energy convergence criterion was set to 10^-6^ Hartree. The DFT-D3(BJ) level correction for dispersion interactions was applied. Structural optimization was performed using the Limited Memory Broyden-Fletcher-Goldfarb-Shannon (LBFGS) optimizer, until the maximum force is below 0.00045 Ry/Bohr (0.011 eV/Å). The charge differential density was calculated and plotted through the software Multiwfn[S6] and Visual Molecular Dynamics (VMD)[S7], respectively. The Gibbs free-energy diagrams were estimated under zero potential (U = 0) by the equation[S8]:

Δ*G*_H_ = Δ*E*_H_ + ΔZPE - *T*Δ*S*	(5)

during which Δ*E*_H_ is the energy change between the reactant and product obtained from DFT calculations, ΔZPE is the change of zero point energy and *T* and Δ*S* represents the temperature and change of entropy, respectively. T = 298.15 K was employed in this case.

**Experimental section**

**Figure S1.** (a-b) TEM images of CDs.

**Figure S2.** FTIR spectra of ZIS, ZIS-NH_2_, ZIS-CDs, and CDs molecules.

**Figure S3.** (a)XPS survey spectra. (b) N 1s of ZIS-CDs samples. (c-f) High-resolution XPS spectra of C 1s, Zn 2p, ln 3d and S 2p for ZIS-NH_2_, CDs and ZIS-CDs.

**Figure S4.** (a-c) BET specific surface area of ZIS, ZIS-NH_2_ and ZIS-CDs. (d-f) The Pore size distribution plot of ZIS, ZIS-NH_2_ and ZIS-CDs.

**Figure S5.** (a) H_2_O_2_ generation rates of different samples under aqueous conditions. (b) H_2_O_2_ generation rates of ZIS-CDs samples in aqueous solutions under air, oxygen, and vacuum atmospheres.

**Figure S6.** (a) Furfuryl alcohol (FFA) test solution in acetonitrile. (b) Reaction solution after 5 h

**Figure S7.** (a)UV−vis spectrum and AQY of H_2_O_2_ production for 5 mg ZIS-CDs. (b) Overview of the recently reported efficiency of photocatalysts in producing H_2_O_2_.

**Figure S8.** (a) Photocatalytic FA production cycle test over ZIS-CDs under 1.1 M FFA. (b) TEM images of ZIS-CDs after the photocatalytic reaction. (c) FTIR spectra of ZIS-CDs after photocatalytic reaction. (d) XRD patterns of ZIS-CDs after photocatalytic reaction.

**Figure S9.** ESR spectra of (a) TEMP-^1^O_2_. (b) DMPO-·OH.

**Figure S10.** (a) Adding dimethylanthracene (DMA) under furfuryl alcohol (FFA) compared to hydrogen peroxide yield. (b) Adding dimethylanthracene (DMA) under benzylamine (BA) compares hydrogen peroxide yield.

**Figure S11.** (a) Koutecky-Levich plots obtained from RRDE measurements. The LSV curves measured on RRDE at different rotation rates for (b) ZIS-CDs with FFA, (c) ZIS-CDs without FFA. (d) Photocatalytic H_2_O_2_ production over ZIS-CDs in 1.1 M FFA solution under different atmospheres (O_2_, O_2_ + air, air, , air + Ar, Ar and N_2_).

**Figure S12.** The ^1^H and ^13^C NMR of furfuryl alcohol (FFA) and furoic acid (FA).

**Figure S13.** Band structure of ZIS, ZIS-NH_2_, ZIS-CDs and CDs.

**Figure S14.** Valence-band XPS spectra of (a) ZIS, (b) ZIS-NH_2_, (c) ZIS-CDs and (d) CDs. Tauc plots of (e) ZIS, (f) ZIS-NH_2_, (g) ZIS-CDs and (h) CDs.

**Figure S15.** UPS spectrum of ZIS-NH_2_, ZIS-CDs

**Table S1.** The details data for calculating AQY of 5 mg ZIS-CDs sample at different wavelengths.

| Wavelength | Photocatalytic H_2_O_2_ production ZIS-CDs | Power |
| --- | --- | --- |
| 400 nm | 32.4 μmol | 0.037 W |
| 420 nm | 31.23 μmol | 0.035 W |
| 500 nm | 32.96 μmol | 0.034 W |
| 600 nm | 5.1 μmol | 0.033 W |

λ = 400 nm:

$$N=\frac{E\lambda}{hc}=\frac{0.037\times3600\times400\times{10}^{-9}}{6.63\times{10}^{-34}\times3\times{10}^{8}}=2.678\times{10}^{20}$$

$$AQY=\frac{2N_{A}n_{H}}{N}\times100\%=\frac{2\times6.02\times{10}^{23}\times\left( (32.4\times{10}^{-6} \right)}{2.678\times{10}^{20}}\times100\%=14.57 \%$$

λ = 420 nm:

$$N=\frac{E\lambda}{hc}=\frac{0.035\times3600\times420\times{10}^{-9}}{6.63\times{10}^{-34}\times3\times{10}^{8}}=2.660\times{10}^{20}$$

$$AQY=\frac{2N_{A}n_{H}}{N}\times100\%=\frac{2\times6.02\times{10}^{23}\times\left( 31.23\times{10}^{-6} \right)}{2.660\times{10}^{20}}\times100\%=13.8 \%$$

λ = 500 nm:

$$N=\frac{E\lambda}{hc}=\frac{0.034\times3600\times500\times{10}^{-9}}{6.63\times{10}^{-34}\times3\times{10}^{8}}=3.077\times{10}^{20}$$

$$AQY=\frac{2N_{A}n_{H}}{N}\times100\%=\frac{2\times6.02\times{10}^{23}\times\left( 32.96\times{10}^{-6} \right)}{3.077\times{10}^{20}}\times100\%=12.9\%$$

λ =600 nm:

$$N=\frac{E\lambda}{hc}=\frac{0.033\times3600\times600\times{10}^{-9}}{6.63\times{10}^{-34}\times3\times{10}^{8}}=2.986\times{10}^{20}$$

$$AQY=\frac{2N_{A}n_{H}}{N}\times100\%=\frac{2\times6.02\times{10}^{23}\times\left( 5.1\times{10}^{-6} \right)}{2.986\times{10}^{20}}\times100\%=2.05\%$$

**Table S2.** Comparison of photocatalytic H_2_O_2_ synthesis performance of ZIS-CDs with that of reported photocatalysts.

| Catalyst | H_2_O_2_ mmolg⁻¹h⁻¹ | AQY (at 400 nm) | SCC | Ref. |
| --- | --- | --- | --- | --- |
| ZIS-CDs | 24 | 14.57% | 1.78% | This work |
| Ni-PuCN | 0.64 | 4.29% | 1.17% | ^[S9]^ |
| BTT-H3 | 1.77 | - | 2.02% | ^[S10]^ |
| In_2_S_3_ | 4.77 | 7.49% | - | ^[S11]^ |
| TiO_2_/MoSₓ-Au | 30.44 | 2.2% | - | ^[S12]^ |
| BCN-FeOOH | 0.34 | 8.36% | 0.95% | ^[S13]^ |
| CNNT-Al | 1.41 | 7.9% | 0.73% | ^[S14]^ |
| TpPa/TpDz | 24.42 | - | 1.34% | ^[S15]^ |
| CD-AQ | 2.5 | - | 0.18% | ^[S16]^ |
| ZC3 | 0.31 | 1.17% |  | ^[S17]^ |
| In_2_S_3_/CdS | 2.09 | 17.73% | - | ^[S18]^ |
| Iso-ZIS | 1.4 | - | - | ^[S19]^ |
| COF/In_2_S_3_ | 5.71 | - | - | ^[S20]^ |
| CNZIS | 1.59 | - | - | ^[S21]^ |
| B-ZnInS | 3.12 | - | 0.59% | ^[S22]^ |
| ZIS/CN | 4.27 | 11.73% | - | ^[S23]^ |
| O-CB/ZnIn_2_S_4_ | 1.98 | - | - | ^[S24]^ |

**Table S3.** Comparison of ZIS-CDs with reported photocatalysts in the photocatalytic conversion of furfuryl alcohol (FFA) and the selectivity of furfural (FAL) to furan acid (FA).

| Catalyst | H_2_O_2_ mmolg⁻¹h⁻¹ | FFA concentration | FA Selectivity | FAL  selectivity | FFA  Conversion | Ref. |
| --- | --- | --- | --- | --- | --- | --- |
| ZIS-CDs | 13 | 16 mM | 100% | 0% | 98% | This Work |
| Cu3-BT-OF | 1.4-2.3 | moderate | 100% | 0% | 99% | ^[S25]^ |
| TiO_2_BTTA ₓ | 0.74 | 2 mM | 96% | 4% | 92% | ^[S26]^ |
| LCP-BT-0 | 0.868 | 4 mM | 100%% | 0% | 95% | ^[S27]^ |

**References**

S1. T. Xiao, L. Wang, K. Li, J. Tang, R. Du, S. Rao, and M. Wu, “Donor‐Acceptor‐Donor Organic Small Molecules as Hole Transfer Vehicle Covalently Coupled Znln_2_S_4_ Nanosheets for Efficient Photocatalytic Hydrogen Evolution,” *Advanced Functional Materials* 35 (2025): 2412644, https://doi.org/10.1002/adfm.202412644.

S2. G. Lippert, J. Hutter, and M. Parrinello, “A hybrid Gaussian and plane wave density functional scheme,” *Molecular Physics.* 92 (1997): 477–487, https://doi.org/10.1080/002689797170220.

S3. J. Vande Vondele, M. Krack, F. Mohamed, M. Parrinello, T. Chassaing, and J. Hutter, “Quickstep: Fast and accurate density functional calculations using a mixed Gaussian and plane waves approach,” *Computer Physics Communications* 167 (2005): 103–128, https://doi.org/[10.1016/j.cpc.2004.12.014](https://www.x-mol.com/paperRedirect/1413839356990525440).

S4. S. Goedecker, M. Teter, and J. Hutter, “Separable dual-space Gaussian pseudopotentials,” *Physical Review B* 54 (1996): 1703–1710, https://doi.org/[10.1103/physrevb.54.1703](https://www.x-mol.com/paperRedirect/1213028483827175427).

S5.	J. P. Perdew, K. Burke, and M. Ernzerhof, “Generalized Gradient Approximation Made Simple,” *Physical Review Letters* 77 (1996): 3865–3868, https://doi.org/[10.1103/physrevlett.77.3865](https://www.x-mol.com/paperRedirect/1212932879669862409).

S6.	T. Lu, F. Chen, “Multiwfn: A multifunctional wavefunction analyzer,” *Journal of Computational Chemistry* 33 (2012): 580–592, https://doi.org/[10.1002/jcc.22885](https://www.x-mol.com/paperRedirect/4626364).

S7.	W. Humphrey, A. Dalke, and K. Schulten, “VMD: Visual molecular dynamics” *Journal of Molecular Graphics* 14 (1996): 33–38, https://doi.org/[10.1016/0263-7855(96)00018-5](https://www.x-mol.com/paperRedirect/1213059590207508486).

S8. M. Bajdich, M. García-Mota, A. Vojvodic, J. K. Nørskov, and A. T. Bell, “Theoretical Investigation of the Activity of Cobalt Oxides for the Electrochemical Oxidation of Water,” *Journal of the American Chemical Society* 135 (2013): 13521–13530, https://doi.org/[10.1021/ja405997s](https://www.x-mol.com/paperRedirect/1773942).

S9.	X. Zhang, H. Su, P. Cui, Y. Cao, Z. Teng, Q. Zhang, Y. Wang, Y. Feng, R. Feng, J. Hou, X. Zhou, P. Ma, H. Hu, K. Wang, C. Wang, L. Gan, Y. Zhao, Q. Liu, T. Zhang, and K. Zheng, “Developing Ni Single-atom Sites in Carbon Nitride for Efficient Photocatalytic H_2_O_2_ production,” *Nature Communications* 14 (2023): 7115, https://doi.org/[10.1038/s41467-023-42887-y](https://www.x-mol.com/paperRedirect/1721995533008785408).

S10. A. Chakraborty, A. Alam, U. Pal, A. Sinha, S. Das, T. Saha-Dasgupta, and P. Pachfule, “Enhancing Photocatalytic Hydrogen Peroxide Generation by Tuning Hydrazone Linkage Density in Covalent Organic Frameworks,” *Nature Communications* 16 (2025): 503, https://doi.org/[10.1038/s41467-025-55894-y](https://www.x-mol.com/paperRedirect/1877426936859787264).

S11. Q. Zhu, J. Su, G. Lin, G. Li, Z. Zhuo, W. Wang, J. Li, and X. Xu, “Surface Indium Vacancies Promote Photocatalytic H_2_O_2_ Production over In_2_S_3_,” *Nature Communications* 16(2025): 10501, https://doi.org/10.1038/s41467-025-65538-w.

S12. X. Zhang, D. Gao, B. Zhu, B. Cheng, J. Yu, and H. Yu, “Enhancing photocatalytic H_2_O_2_ production with Au co-catalysts through electronic structure modification” *Nature Communications* 15 (2024): 3212, https://doi.org/[10.1038/s41467-024-47624-7](https://www.x-mol.com/paperRedirect/1779382978347343872).

S13. P. Liu, T. Liang, Y. Li, Z. Zhang, Z. Li, J. Bian, and L. Jing, “Photocatalytic H_2_O_2_ Production over Boron-Doped g-C_3_N_4_ Containing Coordinatively Unsaturated FeOOH Sites and CoOx Clusters,” *Nature Communications* 15 (2024): 9224, https://doi.org/[10.1038/s41467-024-53482-0](https://www.x-mol.com/paperRedirect/1849983187315138560).

S14. H. Tan, P. Zhou, M. Liu, Y. Gu, W. Chen, H. Guo, J. Zhang, K. Yin, Y. Zhou, C. Shang, Q. Zhang, L. Gu, N. Zhang, J. Ma, Z. Zheng, M. Luo, and S. Guo, “Al–N_3_ Bridge Site Enabling Interlayer Charge Transfer Boosts the Direct Photosynthesis of Hydrogen Peroxide from Water and Air,” *Journal of the American Chemical Society*

146 (2024): 31950–31960, https://doi.org/[10.1021/jacs.4c11471](https://www.x-mol.com/paperRedirect/1854174377728090112).

S15. H. Guo, S. Wang, X. Chen, J. Kou, G. He, Z. Dong, and Y. Yan, “Engineering a Covalent Organic Framework-based Type-II Heterojunction for Enhanced Photocatalytic H_2_O_2_ Synthesis,” *Nature Synthesis* 4 (2025): 1610–1620, https://doi.org/[10.1038/s44160-025-00880-x](https://www.x-mol.com/paperRedirect/1983846089754320896).

S16. M. Gu, D. Y. Lee, J. Mun, D. Kim, H. Cho, B. Kim, W. Kim, G. Lee, B. S. Kim, and H. Kim, “Solar-to-Hydrogen Peroxide Conversion of Photocatalytic Carbon Dots with Anthraquinone: Unveiling the Dual Role of Surface Functionalities,” *Applied Catalysis B: Environmental and Energy* 312 (2022): 121379, https://doi.org/[10.1016/j.apcatb.2022.121379](https://www.x-mol.com/paperRedirect/1513187389685686272).

S17. K. Meng, J. Zhang, B. Cheng, X. Ren, Z. Xia, F. Xu, L. Zhang, and J. Yu, “Plasmonic Near‐Infrared‐Response S‐Scheme ZnO/CuInS_2_ Photocatalyst for H_2_ O_2_ Production Coupled with Glycerin Oxidation,” *Advanced Materials* 36 (2024): 2406460, https://doi.org/10.1038/s44160-025-00880-x.

S18. J. Hu, B. Li, X. Li, T. Yang, X. Yang, J. Qu, Y. Cai, H. Yang, and Z. Lin, “Lattice Match‐Enabled Covalent Heterointerfaces with Built‐in Electric Field for Efficient Hydrogen Peroxide Photosynthesis,” *Advanced Materials* 36 (2024): 2412070. https://doi.org/10.1002/adma.202412070.

S19. X. Ruan, S. Zhao, M. Xu, D. Jiao, J. Leng, G. Fang, D. Meng, Z. Jiang, S. Jin, X. Cui, S. and K. Ravi, “Iso‐Elemental ZnIn_2_ S_4_ /Zn_3_ In_2_ S_6_ Heterojunction with Low Contact Energy Barrier Boosts Artificial Photosynthesis of Hydrogen Peroxide,” *Advanced Energy Materials* 14 (2024): 2401744, https://doi.org/10.1002/aenm. 2024 01744.

S20. J. Qiu, K. Meng, Y. Zhang, B. Cheng, J. Zhang, L. Wang, and J. Yu, “COF/In_2_ S_3_ S‐Scheme Photocatalyst with Enhanced Light Absorption and H_2_ O_2_ ‐Production Activity and fs‐TA Investigation” *Advanced Materials* 36 (2024): 2400288, https://doi.org/10.1002/adma.202400288.

S21. K. Zhang, M. Dan, J. Yang, F. Wu, L. Wang, H. Tang, and Z. Liu, “Surface Energy Mediated Sulfur Vacancy of ZnIn_2_ S_4_ Atomic Layers for Photocatalytic H_2_O_2_ Production,” *Advanced Functional Materials* 33 (2023): 2302964, https://doi.org/10.1002/adfm.202302964.

S22. J. Zhou, Y. Mu, M. Qiao, M. Zhang, S. Yuan, M. Zhang, and T. Lu, “Unlocking One‐Step Two‐Electron Oxygen Reduction via Metalloid Boron‐Modified Zn_3_In_2_S_6_ for Efficient H_2_O_2_ Photosynthesis,” *Angewandte Chemie International Edition* 64 (2025): e202506963, https://doi.org/10.1002/anie.202506963.

S23. Y. Shao, J. Hu, T. Yang, X. Yang, J. Qu, Q. Xu, and C. M. Li, “Significantly Enhanced Photocatalytic in-situ H_2_O_2_ Production and Consumption Activities for Efficient Sterilization by ZnIn_2_S_4_/g-C_3_N_4_ Heterojunction,” *Carbon* 190 (2022): 337–347, https://doi.org/[10.1016/j.apcatb.2023.122780](https://www.x-mol.com/paperRedirect/1650982847783907328).

S24. X. Li, R. Hu, Y. Liu, X. Guo, J. Cheng, Y. Hu, and Y. Chen, “Co-Construction of Oxygen Doping and Van Der Walls Heterojunction in O-CB/ZnIn_2_S_4_ Promoting Photocatalytic Production and Activation of H_2_O_2_ for the Degradation of Antibiotics,” *Journal of Hazardous Materials* 459 (2023): 132187, https://doi.org/[10.1016/j.jhazmat.2023.132187](https://www.x-mol.com/paperRedirect/1686912113501294592).

S25. J. Chang, J. Shi, Q. Li, S. Li, Y. Wang, Y. Chen, F. Yu, S. Li, and Y. Lan, “Regulation of Redox Molecular Junctions in Covalent Organic Frameworks for H_2_O_2_ Photosynthesis Coupled with Biomass Valorization,” *Angewandte Chemie International Edition* 62 (2023): e202303606, https://doi.org/ [10.1002/anie.202303606](https://www.x-mol.com/paperRedirect/1666214533132959744).

S26. Y. Yang, J. Liu, M. Gu, B. Cheng, L. Wang, and J. Yu, “Bifunctional TiO_2_/COF S-scheme Photocatalyst with Enhanced H_2_O_2_ Production and Furoic Acid Synthesis Mechanism,” *Applied Catalysis B: Environment and Energy* 333 (2023): 122780, https://doi.org/[10.1016/j.apcatb.2023.122780](https://www.x-mol.com/paperRedirect/1650982847783907328).

S27. S. Li, R. Ma, C. Tu, W. Zhang, R. Li, Y. Zhao, and K. A. I. Zhang, “Programmed Charge Transfer in Conjugated Polymers with Pendant Benzothiadiazole Acceptor for Simultaneous Photocatalytic H_2_ O_2_ Production and Organic Synthesis,” *Angewandte Chemie International Edition* 64 (2025): e202421040, https://doi.org/[10.1002/anie.202421040](https://www.x-mol.com/paperRedirect/1857217440079245312).
